# Applied methods for matching implementation strategies to determinants: a scoping review of scientific and grey literature, and qualitative exploration of practice experiences

**DOI:** 10.1186/s13012-025-01477-w

**Published:** 2025-12-18

**Authors:** Christiaan Vis, Leti van Bodegom-Vos, Bethany Hipple-Walters, Byron J. Powell, Erwin Ista, Femke van Nassau

**Affiliations:** 1 Department of Public and Occupational Health, Amsterdam UMC, Vrije Universiteit Amsterdam, Amsterdam, The Netherlands; 2 Research Centre for Digital Mental Health Services, Division of Psychiatry, Haukeland University Hospital, Bergen, Norway; 3 Amsterdam Public Health Research Institute, APH, Amsterdam, The Netherlands; 4 Department of Biomedical Data Sciences, Section Medical Decision Making, Leiden University Medical Center, Leiden, the Netherlands; 5 Centre for Implementation, Trimbos Institute, Utrecht, the Netherlands; 6 Center for Mental Health Services Research, George Warren Brown School of Social Work, Washington University in St. Louis, St. Louis, MO USA; 7 Division of Infectious Diseases, John T. Milliken Department of Medicine, School of Medicine, Washington University in St. Louis, St. Louis, MO USA; 8 Dissemination and Implementation Science Innovation Research Network, School of Public Health, Washington University in St. Louis, St. Louis, MO USA; 9 Department of Internal Medicine, Division of Nursing Science, Erasmus MC, University Medical Center Rotterdam, Rotterdam, the Netherlands; 10 Department of Neonatal and Pediatric Intensive Care, Division of Pediatric Intensive Care, Erasmus MC Sophia Children’s Hospital, University Medical Center Rotterdam, Rotterdam, the Netherlands

## Abstract

**Background:**

Tailored implementation addresses the inherent dynamic complexity and heterogeneous nature of implementation practice. In general, tailored implementation involves setting implementation objectives, identifying determinants, matching strategies to those determinants, and developing an evaluation plan. How matching a specific implementation strategy to a determinant is done remains largely unknown. This study aimed to provide an overview of methods for matching strategies that have been applied in research and practice.

**Methods:**

A scoping review of scientific and grey literature was conducted. A Rapid Assessment Procedure approach guided the design and analysis. Five online scientific bibliographic databases and various Dutch websites were searched for scientific and grey literature reporting applied methods for matching strategies to determinants. In addition, fifteen implementation practitioners in the Netherlands were interviewed to gain insights into how matching is conducted in daily practice. Findings were iteratively triangulated.

**Results:**

Fifty-eight scientific articles and ten grey literature documents were included in the review. All identified methods for matching implementation strategies followed a stepped approach and recommended involving both implementation experts and stakeholders at various stages. Almost all methods were based on existing theories, models, and frameworks, such as Intervention Mapping, Expert Recommendations for Implementing Change, and Behaviour Change Wheel. Nevertheless, detailed instructions for matching strategies to determinants were lacking. Similarly, guidance on identifying and involving stakeholders remained superficial. Interviews indicated that in practice, strategy matching is generally based on previous experience and is non-systematic.

**Conclusions:**

Various methods for matching implementation strategies to determinants are reported in literature and used in practice. However, specific and detailed instructions for matching remain lacking. Methods that balance specificity, flexibility, and pragmatism are needed.

**Supplementary Information:**

The online version contains supplementary material available at 10.1186/s13012-025-01477-w.

Contributions to the literature This study makes several contributions to the literature on implementation science and strategy matching:

- It provides an overview of methods for matching implementation strategies to determinants that are used in research and practice.
- It identifies gaps in existing methods, particularly the lack of detailed instructions and unspecified stakeholder involvement.
- By incorporating practitioner insights, it reveals that real-world matching often relies on experience rather than systematic and evidence-informed approaches.
- The study evaluates theoretical frameworks like Intervention Mapping and the Behaviour Change Wheel, highlighting their limitations in providing practical guidance.
- Ultimately, it calls for empirical research to develop methods that balance specificity with flexibility and pragmatism, offering a clear direction for future research in implementation science.

## Background

Developing effective strategies for implementing evidence-based interventions in routine health care and public health settings is a complex and challenging task. Implementation does not take place in a vacuum, but in an environment in which characteristics and circumstances (i.e. determinants) shape implementation processes and outcomes [1, 2]. To increase the likelihood of implementation success, implementation work (i.e. strategies) needs to be prospectively planned and tailored to the implementation context.

Generally, implementation planning involves setting objectives, identifying determinants to achieving those objectives, and selecting strategies that address the most urgent, problematic or modifiable determinants to achieve the objectives [3–5]. Determinants are factors that may facilitate or hinder the implementation of an evidence-based practice, intervention, or innovation in routine care [6]. Implementation strategies are methods that are designed to enhance the adoption, implementation, and sustainability of a practice, intervention, or innovation [7, 8]. The selection of strategies may be ad hoc or follow a more explicit and systematic process.

Ample studies and reviews of the effectiveness of implementation strategies exist [9], notably in the implementation of guidelines and interventions in primary health care [10, 11], in specialised care settings, and in health care management and policy making [12]. However, these studies are little informative about their strategy selection process [13, 14].

Implementing evidence-based practices is challenging because of the dynamic nature of the context in which implementation takes place. One-size-fits-all solutions do not exist because determinants differ across interventions, settings, and implementation phases, and they change over time [1]. Accordingly, the selection of implementation strategies should be systematic, yet flexible, and guided by evidence-informed approaches to ensure that strategies are linked to the determinants at play [15–17]. Matching strategies to determinants generally involve a systematic method to identify determinants and select an implementation strategy [4, 18]. Matching includes ways to organize work related to the identification of potential strategies, prioritization, and final selection of a strategy in relation to an identified determinant as well as operationalizing the strategy into an actionable work plan.

For example, an implementation team in a community mental health setting wishes to reach an underserved population of young male adults who are at risk for developing common mental disorders (depression, anxiety, insomnia, substance misuse). The implementation object concerns a low intensity internet intervention consisting of three psychoeducation modules. The team searches relevant literature and discusses with social workers to identify barriers to implementing this service. They found that patients are difficult to reach due to social stigma. Various health workers mentioned that awareness might be an issue and that people want to talk about their mental health but are ashamed or do not know how to start a conversation. Therefore, the team decided to start an awareness campaign targeting local sports clubs and pubs focussing on normalising mental health issues. This was decided in regular team meetings when members reported back from their conversations with the health care workers. Various materials are developed together with social health workers and owners running the sports clubs and pubs.

There are several frameworks and tools available that implementers can use for selecting and developing implementation strategies [19]. In each of these approaches, either as an explicit step or implicitly in the process, one or more implementation strategies must be matched to one or more determinants. For example, Implementation Mapping [20] guides implementers through a process of identifying change objectives and select associated behavioural change techniques. The CFIR-ERIC Implementation Strategy Matching Tool [21] provides suggestions based on expert-opinion for implementation strategies that are mapped to determinants, and the ItFits-toolkit [22] provides evidence-based online step-by-step guidance for identifying and selecting barriers and strategies, and developing an actionable implementation work plan.

Whereas implementers in practice require specific instructions and materials that resonate with their local tasks and observations, frameworks by definition are based on abstract summaries of multiple implementation trajectories to describe common processes and factors. General applicability is prioritised over specificity in most frameworks (and theories and models). Often, details about matching are not reported, and as a result, frameworks aimed to consolidate knowledge often lack specific criteria and guidance by which the matching is done. To complement existing frameworks and contribute to a basic understanding of matching, this study aimed to synthesise relevant literature and practical knowledge about methods for matching implementation strategies that have been used in research and practice.

## Methods

We conducted (1) a scoping review of international scientific and Dutch grey literature, and (2) semi-structured interviews with implementation practitioners working in the Netherlands. A Rapid Assessment Procedure (RAP, [23, 24]) was used to guide the design and analysis. RAP involves team-based qualitative research using standardised data analysis and supplementary data collection. In our RAP approach, predefined data sheets were used to structure data collection. Within this approach, findings were iteratively triangulated.

### Literature review

A scoping review of scientific literature and Dutch grey literature was performed to obtain an overview of methods used in research to match determinants to implementation strategies.

The databases Medline (medical), Embase (medical), PsycInfo (psychology), CINAHL (nursing), and Web of Science (all sciences) were searched until December 2022 for methods of matching determinants to implementation strategies and their use in implementation processes in healthcare practice. The search strategy is included in Appendix 1. The search string included indexed terms and synonyms for implementation strategies, determinants, and matching. The search string was developed in consultation with a trained librarian.

To complement the findings from the scientific literature review with information about matching methods applied in Dutch implementation practice, relevant publicly accessible grey literature was also searched for methods of matching determinants to implementation strategies. Grey literature was manually collected from online information sources listed in Appendix 1. The sources were searched for documents using the following keywords: implementation strategy or activity, matching determinants or barriers and facilitators, selecting implementation strategies, and implementation methods. Grey literature is, by definition, not standardised in terms of structure and content, which limits relevance and comparability. Each document was therefore manually checked and included in the analysis if it discussed methods for developing implementation strategies in public health and hospital care.

The search strategy, data cleaning, and selection of articles and documents were carried out separately by two research assistants using team-based review software (Covidence). Differences were discussed with a third researcher (CV) to finalise the selection. Articles were eligible for inclusion in the scoping review if they fulfilled the following inclusion criteria:

- The main topic was the implementation of an evidence-based or evidence-informed practice, intervention, or innovation.
- Sufficient details were provided about the methods for matching implementation strategies to identified determinants. Articles that only superficially described the development or selection of an implementation strategy were excluded.
- The setting of the implementation project or process was required to be public health or healthcare.
- Peer-reviewed and written in English or Dutch.

Literature reviews and conference proceedings were excluded as were study protocols when outcome papers were available.

Following the principles of RAP, the research assistants extracted data from the included articles and documents using a standardized data extraction form.

### Semi structured interviews

To promote professional implementation practice, public policy makers, funders, and health care practice have invested in the infrastructure to bring research into practice in the Netherlands. This is done through various means, including grant programming, organising partnerships between research and practice, and investing in the Netherlands Implementation Collaborative (NIC). Recently, the Netherlands were planning to formalise the education of implementation specialists and care professionals through a specialised training program, the articulation of an implementation research agenda, and the establishment of a professional association.

Semi-structured interviews were conducted with various implementation practitioners in the Netherlands to gather information on the methods used to match strategies to determinants in Dutch implementation practice. Eligible interviewees were identified from major networks and organisations in the Netherlands that are known to engage in implementation practice. These organisations represented health professionals and professional associations, implementation practitioners and experts, policy and funding, and knowledge centres and knowledge intermediaries. Because of the expected diversity in functions and titles in implementation practice, we sampled individuals with experience in leading, coordinating, or carrying out implementation work. This included professional functions such as change management, quality management, knowledge transfer, valorisation, and innovation management.

The interviews were semi-structured to ensure all relevant topics were covered while allowing room to ask follow-up questions and seek broader input from the interviewees. The topic guide was developed by the working group (CV, LBV, BHW, EI, and FvN) and is included in Appendix 2. The interviews were conducted online and in Dutch and took approximately 45–60 min. The interviews were conducted in January—March 2023 by members of the working group (CV, LBV, EI, FvN). Following the RAP approach, interviews were not recorded or transcribed. Instead, structured note reports were prepared and used in the analysis by the working group.

### Analysis

Following RAP, triangulation of different sources (literature review and interviews) and team-based data collection and analysis (working group) were central to analytical approach [25, 26]. The information retrieved was sorted and interpreted through an iterative process of reading, reviewing, and structuring observations. In this process, pre-structured literature review data sheets and interview notes were used to inventory the information retrieved. We then used thematic content analysis to further explore patterns in the data. In the working group (CV, FvN, EI, LvB), we periodically took a step back to allow for reflection, re-orientation, and reformulation of the findings. This resulted in descriptive narrative findings about methods for matching implementation strategies to determinants described in literature and used in practice, and the differences between them. These findings were subsequently clarified and enriched through discussion with the wider team of co-authors. Descriptive statistics were applied wherever possible.

## Results

**Fig. 1:**
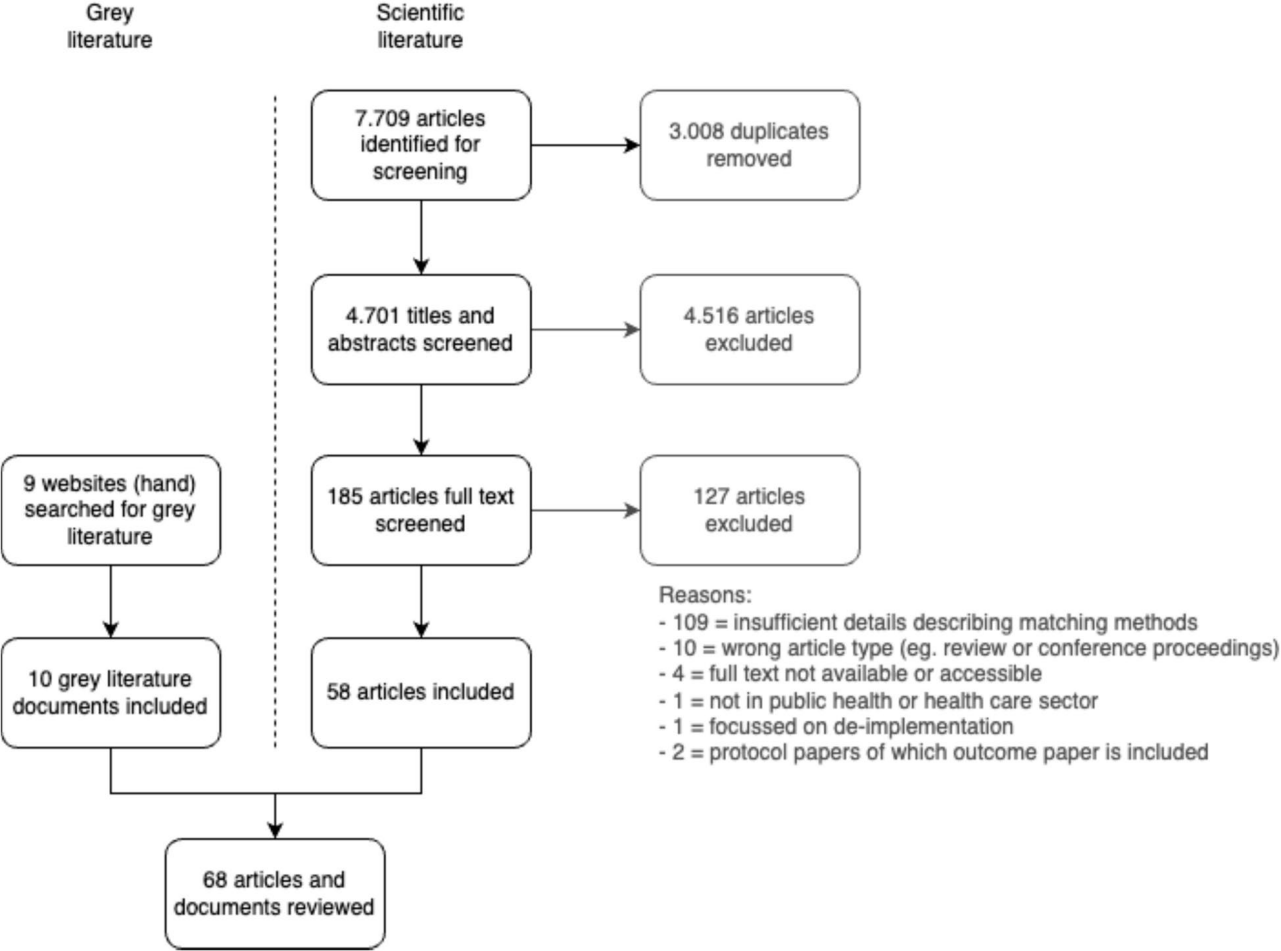
PRISMA flow diagram of the screening and selection process. Grey literature was hand-searched and limited to Dutch websites only. Articles could be excluded based on one or more criteria

The literature search yielded 7709 hits in the scientific literature and 10 grey literature documents (see Fig. 1). After screening a total of 58 scientific articles and 10 grey literature documents were included in the analysis (references are included in Appendix 3).

### General characteristics

More than half of the articles found in the scientific literature (*n* = 33, 57%) focused on implementing a behavioural intervention aimed at treating clinical conditions (*n* = 23, 39%) or lifestyle behaviours (*n* = 8, 14%). Over a quarter (*n* = 16, 28%) focused on implementing a guideline or clinical procedure, and 16% (*n* = 9) combined both a guideline and a clinical intervention. Half (*n* = 29, 50%) of the articles described studies that were conducted in primary care, 22% (*n* = 13) in specialised hospital care, 26% (*n* = 16) in community and public health settings, including prevention and rehabilitation institutions and preschool childcare facilities. Appendix 3 includes an overview of the general characteristics of the studies described in the included articles. The documents found in grey literature either focused on implementing practice guidelines (*n* = 5) or were generic when it comes to the implementation object (*n* = 5).

### Steps

All articles included a stepped approach to developing an implementation plan. This ranged from 2 to 10 steps, with 4 steps most common: 1) scoping the problem and defining the desired target behaviour or situation, 2) identification of facilitating and hindering factors (i.e. determinants) in reaching the desired target outcome and solving the problem, 3) actual matching of strategies to address the targeted determinants, and 4) developing a plan for evaluating implementation outcomes and monitoring progress toward reaching set objectives. In the grey literature, we found a similar pattern. Often, these four steps were complemented by a step focusing on identifying relevant stakeholders. Most of these grey literature documents provided generic advice, illustrated with experience-based examples or best practices. A few methods included worksheets to operationalise the various steps.

### Matching in scientific literature

**Fig. 2:**
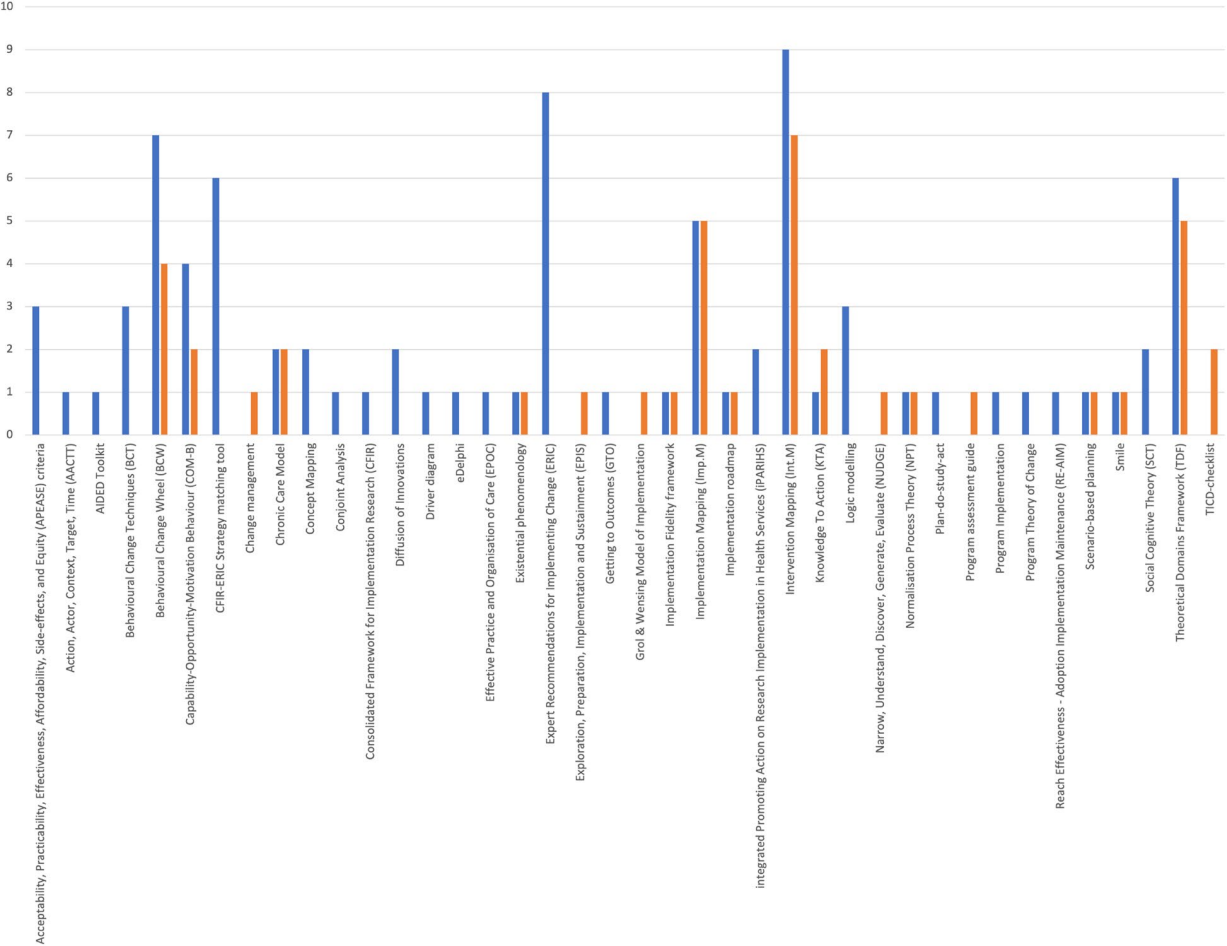
Overview of the frameworks that were used for identifying determinants (orange) and matching strategies to determinants (blue). The frameworks are on the x-axis. A cumulative count is on the y-axis

All reported methods for matching strategies to determinants were based on existing theories, models, or frameworks (from here on referred to: frameworks) of determinants and implementation strategies. Figure 2 provides an overview of the frequency a particular framework was used for matching strategies to determinants. The frameworks used for identifying determinants, i.e. the step before matching, are also included in the figure. Although the frameworks included have different purposes [19], the basis for and way they were used was not appraised in this study.

For identifying determinants, i.e., the step prior to selecting a strategy, 47 (81%) reported using an existing framework. The most frequently used were (*n* = 7) Intervention Mapping [27], (*n* = 6) Theoretical Domains Framework (TDF) [28], and (*n* = 6) Implementation Mapping [20]. Eleven of the 58 (19%) articles used only qualitative methods such as interviews, focus groups, a literature review, previous experience, or a survey to identify determinants and did not utilize a framework.

For matching implementation strategies to determinants, 48 articles (83%) used an existing framework. Other than pre-existing knowledge of or experience with the framework, no prominent justifications were given for why a certain framework was used. The most frequently used frameworks were Intervention Mapping protocol (*n* = 9, [27]), the taxonomy of strategies from ERIC (*n* = 8, [29]), and Behaviour Change Wheel (BCW, *n* = 7, [30]). The Theoretical Domains Framework (TDF, [17]) and the CFIR-ERIC Strategy Matching Tool [21] were both used six times for matching an implementation strategy. Other frameworks or theories such as Normalization Process Theory (NPT, [31]), RE-AIM [23], iPARIHS [24], Diffusion of Innovations [32], Plan-Do-Study-Act cycles, and Getting To Outcomes (GTO, [33]) were used only once or twice as a basis for selecting implementation strategies. Nine articles (16%) did not use an existing theory or framework for describing the matching strategies process. Consensus-oriented methods, e.g., brainstorming, (group) interviews, and surveys were used relatively frequently to aid the selection of implementation strategies and involve stakeholders in that process. Moreover, relatively often, expert opinions, literature reviews, and the research or implementation team's previous experience were used to select implementation strategies.

### Grey literature

The methods found in the grey literature were partly based on existing approaches and frameworks reported in scientific literature. None of the methods provided detailed instructions for matching strategies. Most were based on the ZonMw implementation roadmap (ZonMw stappenplan [34]). This approach includes checklists, worksheets, and sources of inspiration for developing an implementation plan. Although of generic nature, these materials cover topics such as the role and qualities of the project leader, setting goals and objectives, identifying and analysing target groups and their perspective on implementation and their influence, strengths and weaknesses of the intervention to be implemented, a contextual analysis, a checklist for maintenance, and tips for communicating implementation. It also includes information on choosing an implementation strategy and operationalising a strategy into a work plan by setting tasks, budgets, and planning. The ZonMw implementation roadmap is based on the work of Grol and Wensing [18]. Another example is the Implementation Toolkit Technology in Care by Vilans [35]. This approach is also based on the work by Grol and Wensing [18] in combination with De Caluwe and Vermaak [36]. This is a step-by-step guide containing checks and examples of implementing new technology in care practice for older people. Examples and points of attention range from selecting the technology to be implemented to maintaining new practices, and selecting a strategy.

### External expert versus stakeholder-driven matching

Of the methods found in the scientific literature, 18 (31%) were expert-driven and 7 (12%) were stakeholder-driven. The majority (*n* = 33, 57%) were balanced. Expert-driven strategy matching predominantly relied upon the implementation expertise of researchers, external consultants, or scientific knowledge from literature reviews. Stakeholder-driven matching places the input and involvement of local persons or groups such as healthcare professionals, administrators, patients, and project managers affected by the implementation at the center of the selection process. Which person or group was involved when, to what extent, and in what way varied per method. Without exception, implementation processes were led (i.e., ownership) by a team of researchers.

Frequently, stakeholders were consulted in matching strategies through semi-structured (group) interviews. In addition, the prioritization of potentially relevant strategies was guided by pre-established recommendations, such as APEASE; these recommendations on how to prioritize strategies include considerations of available resources, workability, effects, and acceptability. Additionally, expert opinion-based tools were frequently used to select and prioritise strategies by using, for example, the CFIR-ERIC Strategy Matching Tool [21] and TICD checklist [37]. In some cases, stakeholder involvement was complemented by a literature review carried out by a research team. For identifying determinants, stakeholders were most often involved in brainstorming sessions or through surveys.

#### Matching in practice

In total, 15 implementation practitioners working in the Netherlands were interviewed. The majority (*n* = 12, 80%) of the interviewees were female and were highly educated (*n* = 10, 67% postdoctoral or higher). Forty seven percent (*n* = 7) of the interviewees had over 15 years of work experience and most had multiple roles in their organization, varying from implementation advisor (*n* = 6, 40%) to project manager (*n* = 3, 20%), researcher (*n* = 3, 20%), and policy advisor (*n* = 3, 20%). Most interviewees worked for research and innovation institutes (*n* = 7, 47%) or intermediary or networking organisations (*n* = 4, 27%).

Three main themes were found in the interview data illustrating that that implementation practice is a complex and dynamic endeavour: matching implementation strategies (1) requires flexibility, (2) is mostly based on experience, and (3) depends on engagement of the individuals and organisations involved in the implementation.

### Flexibility

Interviewees indicated that, in general, implementing a change in practice often follows an iterative process with incremental advancements. It is *“a winding road” (B1),* partly due to the complex and dynamic nature of health care as well as practical issues such as staff turnover and resource scarcity. This means that it is not always feasible or even preferable to apply a strict and rigid approach. Flexible approaches are required to prospectively adapt to changing priorities, both driven by internal and external factors [38]. As such, implementation in practice is not necessarily associated with a systematic approach; as one interviewee puts it: *“A systematic practice is not applied. This is a thought error.” (A3).* There seems to be a limited assessment of the current state of affairs, setting measurable objectives, and evaluating outcomes. Or as one interviewer indicated: “*it is often a matter of just getting going” (B3).*

### Experience-based

According to respondents, using an existing method for strategy matching is often done on an ad-hoc basis. In practice, implementers *“don't really go through all kinds of steps and a lot happens subconsciously and from experience” (A4).* Over the years and based on courses, literature, and experience, implementation practitioners report having acquired extensive knowledge and skills related to implementation. This is often used to develop “homegrown” roadmaps and worksheets, and they select implementation strategies based on experience, habits, and preferences. This means that, in practice, implementation often depends on the personal ambition, commitment, skills, and experience of the person driving it. Time constraints and the degree of influence in the implementation process are also important aspects. Here, the issue is that the implementation ultimately leads to cost or time savings, and *"the influence of the business case can be frustrating"* (*A4).* Often, implementation budgets are limited and depend on research- or project-related funding. Partly for these reasons, "*the easiest*" *(A3)* is chosen.

Existing methods for selecting an implementation strategy are mostly applied when projects have adequate resources. An example provided by an interviewee is the analytical learning approach by which, first, a distant change goal is formulated, *"the dot on the horizon" (B1).* Then, through iterative questioning and answering with key stakeholders, the rationale for this objective and the path to it are clarified. Methods such as mind mapping, and the use of structured worksheets can be used to shape this step. Once the objectives are clear, specific actions can then be developed. According to one interviewee, stakeholders then know what to do, and *"it is then just a matter of coordination: who works out the actions and who comes up with a proposal" (B1).* However, in this, interviewees mentioned that the actual selection of actions that match the identified objectives is mostly made based on brainstorming, experience, and personal beliefs, and there is limited use of structural guidance in these processes.

Nevertheless, existing taxonomies to identify barriers and facilitators to select strategies that might address them are used in practice. Respondents noted that frameworks by Grol & Wensing [18], CFIR [2], Intervention Mapping protocol [27], ZonMw implementation roadmap [34], and design principles such as the Double Diamond model [39] were used as tools to implement interventions in health care practices. Such models can be used *“to the letter” (C1),* or as a source of inspiration, applying only the principles, or to obtain ideas. Nevertheless, linking strategies to determinants remains *“tricky” (B3).* The operationalisation of a strategy into an actionable work plan was often regarded difficult by the interviewees as *“the limitation of taxonomies is that they are not yet very concrete in options as to what such a strategy should look like” (B3).* Also, the evidence base of the causal relationship between strategy and determinant is unclear, not the least because of the context dependencies and dynamic nature of implementation practice.

### Engagement

A possible aspect in implementation in general and in matching strategies specifically relates to engagement of the individuals and organisations involved in the implementation. It was mentioned several times by the interviewees, that as an implementation practitioner, it is important to have a good connection with the place where the implementation takes place: *“what is going on there, and therefore what is appropriate in this specific place” (B2).* As a first step, many respondents mentioned starting with *“talking” (A1*) with the various stakeholders and proceeding as required and feasible in the local context. Likewise, it is important that the people affected by the change are involved in the implementation and its preparation. A directive approach often does not work, as illustrated by one interviewee: *“Top-down rolling out does not work, but you can inspire and contribute ideas from the top […] Together you will get further. You may go slower but you get further in the end.” (B2).* Furthermore, a recommendation made by some interviewees was to educate the practice: *“A lot of people don't know. And you can't blame them.” (A3).* This goes beyond the systematic or evidence-informed development of an implementation strategy: *“[we] need more than a plan; [we] need more vibrancy.” (A3).*

From a policy and funders’ perspective, there is increased attention to *“shared responsibility” (A2)* in improving research use in practice through effective implementation. Funding agencies take a more active role and move the focus from identifying problems to the logic in choosing implementation strategies and the scientific underpinning thereof. Research institutes and intermediary organisations, however, take a facilitating role and emphasise that *“implementation is up to the field” (C2).* However, knowledge implementation is complex and, in the Netherlands, severely hampered by the limited availability of expert knowledge to serve practice. It is often *“the usual suspects” (A2)* who are involved and who become easily over-demanded.

## Discussion

This study provides two perspectives on methods for matching implementation strategies: one based on research using (scientific and grey) literature, and one based on implementation practice using interview data. Generally, matching is part of an implementation strategy selection process that includes setting implementation objectives, identifying determinants, matching strategies to address determinants, and an evaluation plan. Most of the reviewed literature (both scientific and grey) discuss such approaches. However, we found that none provides comprehensive, detailed guidance for the actual identification, prioritisation, selection, and operationalisation of strategies that are matched to address specific determinants. Detailed insight into methods and criteria by which strategies are matched to determinants is lacking. Current approaches to matching rely on expert opinion or stakeholder input and mostly provide only superficial guidance. These findings either reflect the actual current state of the art in matching, poor reporting practices, or most likely, a combination of both.

In practice, on the other hand, approaches to matching are applied flexibly and are often pragmatic and experience based. Although guidance documents are available, insights from implementation research seem to inform implementation practice in a limited way. This can lead to choosing the obvious implementation strategies with which implementers have experience, such as educational strategies, and do not necessarily address determinants of implementation. Limitations in resources, unfamiliarity with other strategies or approaches, as well as the complex and dynamic nature of practice, might inhibit applying insights from research.

The lack of guidance for matching implementation strategies to determinants has been recognised by others as well. For example, Beidas and colleagues [40] conclude in their reflection on the current state of the art in the field of implementation research that while the basic steps of implementation strategy development are documented well, the process of selecting implementation strategies and tailoring them to address identified determinants is not. Partly, this could be due to the lengthy and complex processes often involving extensive negotiation of the interests of the various stakeholders involved. As such, it requires balancing achieving rigour and practical feasibility in methods for matching implementation strategies [40]. Similarly, Balis and Houghtaling debated recommendations for linking strategies to determinants in public health [41]. Recent work by Balis et al. proposed the ISAC Match tool detailing guidance partnerships of researchers and practitioners for strategy selection, adaptation, evaluation, and decision making [42]. Other examples include concept mapping, group model building, conjoint analysis, and implementation mapping [15], which we have identified in the literature as well. These methods have a participatory focus that revolve around active involvement of experts and stakeholders. This requires specific skills and evidence of their value in heterogeneous settings might be limited [41]. Nevertheless, and while respecting the need for better methods, this should not be understood as that matching will become easy or formulaic.

Besides the relevance of selecting those strategies that are known to be effective, we found two other aspects to be part of a matching process: (1) involving stakeholders and (2) process management, i.e. a stepped approach. The matching methods reported in the literature and used in practice facilitate the involvement of both experts and stakeholders. However, the manner, extent, roles, and responsibilities of experts and stakeholders vary. Involvement of both implementation expertise and local stakeholders is relevant because determinants, and thus implementation strategies, are context and time-dependent and thus differ with context and change over time [1].

Generally, it is common practice to involve stakeholders in implementation [43]. Stakeholder involvement can consist of several elements, including systematic identification of individuals and organisations, different ways of engaging stakeholders, and stakeholder-by-stakeholder mapping of interests and influence [44]. Our analysis showed that none of the methods, from the literature nor in Dutch implementation practice, explicitly addresses stakeholder identification and engagement. Qualifications and procedures for involving experts are not specified in the analysed literature.

A further similarity between the scientific literature and the practice documentation is that all approaches propose a step-by-step approach that includes strategy matching as a distinct step. However, the degree of detailing of the step that involves matching is limited. When this step is specified, it is mainly by examples or by referring to frameworks and taxonomies for inspiration. Nevertheless, the approaches for the development of an implementation strategy aid in managing complexity by breaking down the process of identification, selection, and development into manageable tasks.

### Recommendations

Although the practice of selecting implementation strategies is often under time pressure, stepped approaches can ensure that emerging issues in matching implementation strategies can be discussed and reflected upon in a timely manner. While this may be at odds with the prevailing time constraints, repetitive and prospective reflection might lead to better-aligned implementation strategies. Nevertheless, we found that current methods do not provide comprehensive, detailed guidance for the actual identification, prioritisation, selection, and operationalisation of strategies that are matched to address specific determinants. A key recommendation, therefore, is to develop practical guidance for matching strategies to determinants that include (sets of) criteria to facilitate decision making in changing circumstances and unique contexts. Although evidence is sparse, methods for tailored implementation seem worthwhile to further explore [22, 45]. Such guidance should balance flexibility and pragmatism with standardisation and structure to cater requirements of specific situations, stakeholders, and contextual factors. A further key recommendation is to emphasise theory-based approaches in matching strategies that address underlying patterns such as habits, organisational culture, or procedural barriers. Reflection, the step-by-step approach, and use of implementation theories, models, and frameworks might prevent lock-in that might emerge from experience-based approaches. Moreover, future research should propose the key ingredients of matching methods that, ideally, are based on empirical research in combination with expert opinion.

The fact that detailed guidance on stakeholder involvement is largely lacking in the methods we found contradicts the common understanding of the importance of stakeholder involvement in strategy matching. We suggest exploring in detail how stakeholder involvement can be shaped in strategy matching and include instructions for effective stakeholder identification and consultation.

Some initial work has been conducted [44, 46], existing approaches such as participatory action research or co-creation from organisational studies may be of value for implementation projects. The dynamic nature of implementation practice and required flexibility to involve the right stakeholder at the right time and in the right way should receive notable attention.

Moreover, new insights should serve not only the scientific community but also implementation practitioners. This requires improving accessibility and usability of knowledge on matching methods, processes, and outcomes. User-friendly tools must be made available for implementation practitioners that enable them to develop strategies in an effective and efficient manner. Together with implementation practitioners, the functionalities of such guidance should be explicitly defined. Subsequently, evidence should be generated of the types of decisions the tools can guide implementation practitioners through.

### Strengths and limitations

This study sought to provide an overview of applied methods for matching strategies to determinants. The complementarity of methods that were used is a notable strength. Although the review followed a Rapid Assessment Procedure, the yield was considerable and broad. Similarly, the semi-structured interviews allowed us to gain insight into the matching in practice. By combining a systematic review of scientific and grey literature and by asking implementation practitioners in the field about their experiences, we could contrast and deepen the findings. However, there are some limitations that need to be considered.

First it must be note that the topic has received limited scientific attention in research and practice. Acknowledging the search date (December 2022), this is underlined by the fact that the oldest included article in our review is from 2012, and the bulk of the articles were published in the last five years. Secondly, this study did not assess whether the identified evidence-based interventions include embedded determinant and implementation strategy guidance within their dissemination materials. Well-prepared interventions often embed supports to promote fidelity, suggesting that implementation efforts may reinforce rather than augment existing strategies [5]. Nevertheless, such details are rarely documented and would require studying specific literature, which is beyond this study’s scope. Another limitation is that the broad search structure yielded a heterogeneous corpus of literature. The identified studies varied in, for example, implementation objects and context. Consequently, it posed a challenge to gain a shared understanding of the concept of matching applied in the articles to be able to screen and select papers as systematically and objectively as possible. By working as a team, screening articles for inclusion with three researchers and conducting various checks on the extracted information, the quality of the review process was optimised as much as possible. Finaly, this study is that it partially focused on implementation practice in the Dutch health care setting. Although the scoping review was open to international (English language) peer-reviewed articles, the review of grey literature and interviews with implementers focused on Dutch organisations and were conducted with implementers working in the Netherlands. Supplementing the perspectives of the scientific community with those of the implementation practice community is a strength. However, the practice view is limited to Dutch experiences only.

## Conclusions

Various methods to aid in matching implementation strategies to determinants used in research and practice. Almost all are based on existing theories, frameworks, and taxonomies, of which Intervention Mapping, ERIC, and Behaviour Change Wheel were mentioned most often. All methods include a stepped approach, breaking down the required work to develop a strategy. All involve both experts and stakeholders in the various steps. In practice, however, often implementation strategies are matched to determinants based on experience. Similarly, guidance in stakeholder identification and engagement is absent in the methods analysed in this study. The accessibility and usability of knowledge about matching implementation strategies to determinants should be translated into practical, workable tools that can inform implementation practice. Empirical evidence is required to develop specific, detailed yet flexible guidance on matching strategies to determinants and on stakeholder involvement in those processes.

## Supplementary Information

Supplementary Material 1.

Supplementary Material 2.

Supplementary Material 3.

## Data Availability

All data generated or analysed during this study are included in this published article and its supplementary information files.
