# Supplementary material for: Applied methods for matching implementation strategies to determinants: a scoping review of scientific and grey literature, and qualitative exploration of practice experiences
Source: Implement Sci. 2025 Dec 18;21:14. doi: 10.1186/s13012-025-01477-w (PMC12911165; doi:10.1186/s13012-025-01477-w)
Supplement: Supplementary file 1 — Supplementary Material 1. [file 13012_2025_1477_MOESM1_ESM.docx]

# Appendix 1

Contents:

- Search strategy for systematic review
- Sources for searching Dutch grey literature

# Search strategy

December 9th 2022

## PubMed

### Search strings

**# Implementation strategy**

"Implementation Science"[Mesh] OR "Health Plan Implementation"[Mesh] OR “implementation science*”[tiab] OR "implementation strateg*"[tiab] OR "implementation intervention*"[tiab] OR "implementation action*"[tiab] OR "implementation plan*"[tiab] OR "implementation techni*"[tiab] OR “health plan implementation”[tiab] OR “implementation research”[tiab] OR “implementation scientific research”[tiab] OR “health care policy”[tiab]

**# Determinants**

"Decision Theory"[Mesh:NoExp] OR determinant*[tiab] OR barrier*[tiab] OR “hindering factor*”[tiab] OR “implementation factor*”[tiab] OR enabler*[tiab] OR facilitat*[tiab]

**# Matching**

“knowledge translation”[tiab] OR “adopt*”[tiab] OR “scaling up”[tiab] OR “scaling out”[tiab] OR Matching[tiab] OR linking[tiab] OR selecting[tiab] OR tailoring[tiab] OR developing[tiab] OR development[tiab] OR uptake[tiab] OR unfolding[tiab] OR translating[tiab]

**# Setting**

"Delivery of Health Care"[Mesh:NoExp] OR "Health Services Research"[Mesh] OR "Health Care Sector"[Mesh] OR "Health Services"[Mesh:NoExp] OR "Patient Care"[Mesh:NoExp] OR "School Health Services"[Mesh] OR “health care”[tiab] OR “health intervention*”[tiab] OR “health care setting*”[tiab] OR “health setting*”[tiab] OR “health service*”[tiab] OR “health sector*”[tiab] OR “school health service*”[tiab] OR “school health care*”[tiab]

### Results 1.752 records

| Search | Query | Results |
| --- | --- | --- |
| #5 | Search: #1 AND #2 AND #3 AND #4 Sort by: Most Recent | [1,752](https://pubmed.ncbi.nlm.nih.gov/?term=%231+AND+%232+AND+%233+AND+%234&sort=date) |
| #4 | Search: "Delivery of Health Care"[Mesh:NoExp] OR "Health Services Research"[Mesh] OR "Health Care Sector"[Mesh] OR "Health Services"[Mesh:NoExp] OR "Patient Care"[Mesh:NoExp] OR "School Health Services"[Mesh] OR "health care"[tiab] OR "health intervention*"[tiab] OR "health care setting*"[tiab] OR "health setting*"[tiab] OR "health service*"[tiab] OR "health sector*"[tiab] OR "school health service*"[tiab] OR "school health care*"[tiab] Sort by: Most Recent | [830,642](https://pubmed.ncbi.nlm.nih.gov/?term=%22Delivery+of+Health+Care%22%5BMesh%3ANoExp%5D+OR+%22Health+Services+Research%22%5BMesh%5D+OR+%22Health+Care+Sector%22%5BMesh%5D+OR+%22Health+Services%22%5BMesh%3ANoExp%5D+OR+%22Patient+Care%22%5BMesh%3ANoExp%5D+OR+%22School+Health+Services%22%5BMesh%5D+OR+%E2%80%9Chealth+care%E2%80%9D%5Btiab%5D+OR+%E2%80%9Chealth+intervention%2A%E2%80%9D%5Btiab%5D+OR+%E2%80%9Chealth+care+setting%2A%E2%80%9D%5Btiab%5D+OR+%E2%80%9Chealth+setting%2A%E2%80%9D%5Btiab%5D+OR+%E2%80%9Chealth+service%2A%E2%80%9D%5Btiab%5D+OR+%E2%80%9Chealth+sector%2A%E2%80%9D%5Btiab%5D+OR+%E2%80%9Cschool+health+service%2A%E2%80%9D%5Btiab%5D+OR+%E2%80%9Cschool+health+care%2A%E2%80%9D%5Btiab%5D&sort=date) |
| #3 | Search: "knowledge translation"[tiab] OR "adopt*"[tiab] OR "scaling up"[tiab] OR "scaling out"[tiab] OR Matching[tiab] OR linking[tiab] OR selecting[tiab] OR tailoring[tiab] OR developing[tiab] OR development[tiab] OR uptake[tiab] OR unfolding[tiab] OR translating[tiab] Sort by: Most Recent | [4,031,096](https://pubmed.ncbi.nlm.nih.gov/?term=%E2%80%9Cknowledge+translation%E2%80%9D%5Btiab%5D+OR+%E2%80%9Cadopt%2A%E2%80%9D%5Btiab%5D+OR+%E2%80%9Cscaling+up%E2%80%9D%5Btiab%5D+OR+%E2%80%9Cscaling+out%E2%80%9D%5Btiab%5D+OR+Matching%5Btiab%5D+OR+linking%5Btiab%5D+OR+selecting%5Btiab%5D+OR+tailoring%5Btiab%5D+OR+developing%5Btiab%5D+OR+development%5Btiab%5D+OR+uptake%5Btiab%5D+OR+unfolding%5Btiab%5D+OR+translating%5Btiab%5D&sort=date) |
| #2 | Search: "Decision Theory"[Mesh:NoExp] OR determinant*[tiab] OR barrier*[tiab] OR "hindering factor*"[tiab] OR "implementation factor*"[tiab] OR enabler*[tiab] OR facilitat*[tiab] Sort by: Most Recent | [1,266,010](https://pubmed.ncbi.nlm.nih.gov/?term=%22Decision+Theory%22%5BMesh%3ANoExp%5D+OR+determinant%2A%5Btiab%5D+OR+barrier%2A%5Btiab%5D+OR+%E2%80%9Chindering+factor%2A%E2%80%9D%5Btiab%5D+OR++%E2%80%9Cimplementation+factor%2A%E2%80%9D%5Btiab%5D+OR+enabler%2A%5Btiab%5D+OR+facilitat%2A%5Btiab%5D&sort=date) |
| #1 | Search: "Implementation Science"[Mesh] OR "Health Plan Implementation"[Mesh] OR "implementation science*"[tiab] OR "implementation strateg*"[tiab] OR "implementation intervention*"[tiab] OR "implementation action*"[tiab] OR "implementation plan*"[tiab] OR "implementation techni*"[tiab] OR "health plan implementation"[tiab] OR "implementation research"[tiab] OR "implementation scientific research"[tiab] OR "health care policy"[tiab] Sort by: Most Recent | [23,197](https://pubmed.ncbi.nlm.nih.gov/?term=%22Implementation+Science%22%5BMesh%5D+OR+%22Health+Plan+Implementation%22%5BMesh%5D+OR+%E2%80%9Cimplementation+science%2A%E2%80%9D%5Btiab%5D+OR+%22implementation+strateg%2A%22%5Btiab%5D+OR+%22implementation+intervention%2A%22%5Btiab%5D+OR+%22implementation+action%2A%22%5Btiab%5D+OR+%22implementation+plan%2A%22%5Btiab%5D+OR+%22implementation+techni%2A%22%5Btiab%5D+OR+%E2%80%9Chealth+plan+implementation%E2%80%9D%5Btiab%5D+OR+%E2%80%9Cimplementation+research%E2%80%9D%5Btiab%5D+OR+%E2%80%9Cimplementation+scientific+research%E2%80%9D%5Btiab%5D+OR+%E2%80%9Chealth+care+policy%E2%80%9D%5Btiab%5D+&sort=date) |

## Embase (Embase.com)

### Search strings

**# Implementation strategy**

'implementation science'/exp OR 'health care planning'/de OR ‘implementation science*’:ab,ti,kw OR ‘implementation strateg*’:ab,ti,kw OR ‘implementation intervention*’:ab,ti,kw OR ‘implementation action*’:ab,ti,kw OR ‘implementation plan*’:ab,ti,kw OR ‘implementation techni*’:ab,ti,kw OR ‘health plan implementation’:ab,ti,kw OR ‘implementation research’:ab,ti,kw OR ‘implementation scientific research’:ab,ti,kw OR ‘health care policy’:ab,ti,kw

**# Determinants**

'decision theory'/exp OR determinant*:ab,ti,kw OR barrier*:ab,ti,kw OR ‘hindering factor*’:ab,ti,kw OR ‘implementation factor*’:ab,ti,kw OR enabler*:ab,ti,kw OR facilitat*:ab,ti,kw

**# Matching**

'knowledge translation'/exp OR ‘knowledge translation’:ab,ti,kw OR ‘translational science*’:ab,ti,kw OR ‘adopt*’:ab,ti,kw OR ‘scaling up’:ab,ti,kw OR ‘scaling out’:ab,ti,kw OR Matching:ab,ti,kw OR linking:ab,ti,kw OR selecting:ab,ti,kw OR tailoring:ab,ti,kw OR developing:ab,ti,kw OR development:ab,ti,kw OR uptake:ab,ti,kw OR unfolding:ab,ti,kw OR translating:ab,ti,kw

**# Setting**

'health care delivery'/de OR 'health services research'/exp OR 'health service'/de OR 'health care'/de OR 'patient care'/de OR 'school health service'/exp OR ‘health care’:ab,ti,kw OR ‘health intervention*’:ab,ti,kw OR ‘health care setting*’:ab,ti,kw OR ‘health setting*’:ab,ti,kw OR ‘health service*’:ab,ti,kw OR ‘health sector*’:ab,ti,kw OR ‘school health service*’:ab,ti,kw OR ‘school health care*’:ab,ti,kw

**# Filters**

('article'/it OR 'article in press'/it OR 'note'/it OR 'short survey'/it)

### Results 2.631 records

| **No.** | **Query** | **Results** |
| --- | --- | --- |
| **#6** | **#5** AND (**'article'**/it OR **'article in press'**/it OR **'note'**/it OR **'short survey'**/it) | **2,631** |
| **#5** | **#1** AND **#2** AND **#3** AND **#4** | **3,641** |
| **#4** | **'health care delivery'**/de OR **'health services research'**/exp OR **'health service'**/de OR **'health care'**/de OR **'patient care'**/de OR **'school health service'**/exp OR **'health care'**:ab,ti,kw OR **'health intervention*'**:ab,ti,kw OR **'health care setting*'**:ab,ti,kw OR **'health setting*'**:ab,ti,kw OR **'health service*'**:ab,ti,kw OR **'health sector*'**:ab,ti,kw OR **'school health service*'**:ab,ti,kw OR **'school health care*'**:ab,ti,kw | **1,336,828** |
| **#3** | **'knowledge translation'**/exp OR **'knowledge translation'**:ab,ti,kw OR **'translational science*'**:ab,ti,kw OR **'adopt*'**:ab,ti,kw OR **'scaling up'**:ab,ti,kw OR **'scaling out'**:ab,ti,kw OR **matching**:ab,ti,kw OR **linking**:ab,ti,kw OR **selecting**:ab,ti,kw OR **tailoring**:ab,ti,kw OR **developing**:ab,ti,kw OR **development**:ab,ti,kw OR **uptake**:ab,ti,kw OR **unfolding**:ab,ti,kw OR **translating**:ab,ti,kw | **5,082,890** |
| **#2** | **'decision theory'**/exp OR **determinant***:ab,ti,kw OR **barrier***:ab,ti,kw OR **'hindering factor*'**:ab,ti,kw OR **'implementation factor*'**:ab,ti,kw OR **enabler***:ab,ti,kw OR **facilitat***:ab,ti,kw | **1,554,463** |
| **#1** | **'implementation science'**/exp OR **'health care planning'**/de OR **'implementation science*'**:ab,ti,kw OR **'implementation strateg*'**:ab,ti,kw OR **'implementation intervention*'**:ab,ti,kw OR **'implementation action*'**:ab,ti,kw OR **'implementation plan*'**:ab,ti,kw OR **'implementation techni*'**:ab,ti,kw OR **'health plan implementation'**:ab,ti,kw OR **'implementation research'**:ab,ti,kw OR **'implementation scientific research'**:ab,ti,kw OR **'health care policy'**:ab,ti,kw | **129,713** |

## APA PsycInfo (EBSCO)

### Search strings

**# Implementation strategy**

DE "Health Care Policy" OR DE "Policy Making" OR TI(“implementation science*” OR "implementation strateg*" OR "implementation intervention*" OR "implementation action*" OR "implementation plan*" OR "implementation techni*" OR “health plan implementation” OR “implementation research” OR implementation scientific research” OR “health care policy”) OR AB(“implementation science*” OR "implementation strateg*" OR "implementation intervention*" OR "implementation action*" OR "implementation plan*" OR "implementation techni*" OR “health plan implementation” OR “implementation research” OR implementation scientific research” OR “health care policy”) OR KW(“implementation science*” OR "implementation strateg*" OR "implementation intervention*" OR "implementation action*" OR "implementation plan*" OR "implementation techni*" OR “health plan implementation” OR “implementation research” OR implementation scientific research” OR “health care policy”)

**# Determinants**

DE "Decision Theory" OR DE "Treatment Barriers" OR TI(determinant* OR barrier* OR “hindering factor*” OR “implementation factor*” OR enabler* OR facilitat*) OR AB(determinant* OR barrier* OR “hindering factor*” OR “implementation factor*” OR enabler* OR facilitat*) OR KW(determinant* OR barrier* OR “hindering factor*” OR “implementation factor*” OR enabler* OR facilitat*)

**# Matching**

DE "Knowledge Transfer" OR TI(“knowledge translation” OR “translational science*” OR “adopt*” OR “scaling up” OR “scaling out” OR Matching OR linking OR selecting OR tailoring OR developing OR development OR uptake OR unfolding OR translating) OR AB(“knowledge translation” OR “translational science*” OR “adopt*” OR “scaling up” OR “scaling out” OR Matching OR linking OR selecting OR tailoring OR developing OR development OR uptake OR unfolding OR translating) OR KW(“knowledge translation” OR “translational science*” OR “adopt*” OR “scaling up” OR “scaling out” OR Matching OR linking OR selecting OR tailoring OR developing OR development OR uptake OR unfolding OR translating)

**# Setting**

DE "Health Care Services" OR DE "Health Care Delivery" OR TI(“health care” OR “health intervention*” OR “health care setting*” OR “health setting*” OR “health service*” OR “health sector*” OR “school health service*” OR “school health care*”) OR AB(“health care” OR “health intervention*” OR “health care setting*” OR “health setting*” OR “health service*” OR “health sector*” OR “school health service*” OR “school health care*”) OR KW(“health care” OR “health intervention*” OR “health care setting*” OR “health setting*” OR “health service*” OR “health sector*” OR “school health service*” OR “school health care*”)

### Results 567 records

| **#** | **Query** | **Limiters** | **Results** |
| --- | --- | --- | --- |
| S6 | S1 AND S2 AND S3 AND S4 | Limiters –  Academic journals | 567 |
| S5 | S1 AND S2 AND S3 AND S4 | Search modes - Boolean/Phrase | 659 |
| S4 | DE "Health Care Services" OR DE "Health Care Delivery" OR TI(“health care” OR “health intervention*” OR “health care setting*” OR “health setting*” OR “health service*” OR “health sector*” OR “school health service*” OR “school health care*”) OR AB(“health care” OR “health intervention*” OR “health care setting*” OR “health setting*” OR “health service*” OR “health sector*” OR “school health service*” OR “school health care*”) OR KW(“health care” OR “health intervention*” OR “health care setting*” OR “health setting*” OR “health service*” OR “health sector*” OR “school health service*” OR “school health care*”) | Search modes - Boolean/Phrase | 209,162 |
| S3 | DE "Knowledge Transfer" OR TI(“knowledge translation” OR “translational science*” OR “adopt*” OR “scaling up” OR “scaling out” OR Matching OR linking OR selecting OR tailoring OR developing OR development OR uptake OR unfolding OR translating) OR AB(“knowledge translation” OR “translational science*” OR “adopt*” OR “scaling up” OR “scaling out” OR Matching OR linking OR selecting OR tailoring OR developing OR development OR uptake OR unfolding OR translating) OR KW(“knowledge translation” OR “translational science*” OR “adopt*” OR “scaling up” OR “scaling out” OR Matching OR linking OR selecting OR tailoring OR developing OR development OR uptake OR unfolding OR translating) | Search modes - Boolean/Phrase | 1,036,677 |
| S2 | DE "Decision Theory" OR DE "Treatment Barriers" OR TI(determinant* OR barrier* OR “hindering factor*” OR “implementation factor*” OR enabler* OR facilitat*) OR AB(determinant* OR barrier* OR “hindering factor*” OR “implementation factor*” OR enabler* OR facilitat*) OR KW(determinant* OR barrier* OR “hindering factor*” OR “implementation factor*” OR enabler* OR facilitat*) | Search modes - Boolean/Phrase | 326,694 |
| S1 | DE "Health Care Policy" OR DE "Policy Making" OR TI(“implementation science*” OR "implementation strateg*" OR "implementation intervention*" OR "implementation action*" OR "implementation plan*" OR "implementation techni*" OR “health plan implementation” OR “implementation research” OR implementation scientific research” OR “health care policy”) OR AB(“implementation science*” OR "implementation strateg*" OR "implementation intervention*" OR "implementation action*" OR "implementation plan*" OR "implementation techni*" OR “health plan implementation” OR “implementation research” OR implementation scientific research” OR “health care policy”) OR KW(“implementation science*” OR "implementation strateg*" OR "implementation intervention*" OR "implementation action*" OR "implementation plan*" OR "implementation techni*" OR “health plan implementation” OR “implementation research” OR implementation scientific research” OR “health care policy”) | Search modes - Boolean/Phrase | 42,124 |

## Cinahl (EBSCO)

### Searchstrings

**# Implementation strategy**

(MH "Implementation Science") OR TI(“implementation science*” OR "implementation strateg*" OR "implementation intervention*" OR "implementation action*" OR "implementation plan*" OR "implementation techni*" OR “health plan implementation” OR “implementation research” OR “implementation scientific research” OR “health care policy”) OR AB(“implementation science*” OR "implementation strateg*" OR "implementation intervention*" OR "implementation action*" OR "implementation plan*" OR "implementation techni*" OR “health plan implementation” OR “implementation research” OR “implementation scientific research” OR “health care policy”) OR SU(“implementation science*” OR "implementation strateg*" OR "implementation intervention*" OR "implementation action*" OR "implementation plan*" OR "implementation techni*" OR “health plan implementation” OR “implementation research” OR “implementation scientific research” OR “health care policy”)

**# Determinants**

TI(determinant* OR barrier* OR “hindering factor*” OR “implementation factor*” OR enabler* OR facilitat*) OR AB(determinant* OR barrier* OR “hindering factor*” OR “implementation factor*” OR enabler* OR facilitat*) OR SU(determinant* OR barrier* OR “hindering factor*” OR “implementation factor*” OR enabler* OR facilitat*)

**# Matching**

TI(“knowledge translation” OR “adopt*” OR “scaling up” OR “scaling out” OR Matching OR linking OR selecting OR tailoring OR developing OR development OR uptake OR unfolding OR translating) OR AB(“knowledge translation” OR “adopt*” OR “scaling up” OR “scaling out” OR Matching OR linking OR selecting OR tailoring OR developing OR development OR uptake OR unfolding OR translating) OR SU(“knowledge translation” OR “adopt*” OR “scaling up” OR “scaling out” OR Matching OR linking OR selecting OR tailoring OR developing OR development OR uptake OR unfolding OR translating)

**# Setting**

(MM "Healthcare Disparities") OR (MM "Health Care Delivery, Integrated") OR (MH "Health Care Delivery") OR (MH "Health Services Research") OR (MM "Policy Studies") OR (MM "Methodological Research") OR (MM "Usability Study") OR (MM "Quality of Care Research") OR (MH "Outcomes Research") OR (MH "Health Services") OR (MH "Patient Care") OR (MH "School Health Services") OR (MM "School Health Education") OR (MM "School Health Nursing") OR (MM "School Mental Health Services") OR TI( “health care” OR “health intervention*” OR “health care setting*” OR “health setting*” OR “health service*” OR “health sector*” OR “school health service*” OR “school health care*”) OR AB( “health care” OR “health intervention*” OR “health care setting*” OR “health setting*” OR “health service*” OR “health sector*” OR “school health service*” OR “school health care*”) OR SU( “health care” OR “health intervention*” OR “health care setting*” OR “health setting*” OR “health service*” OR “health sector*” OR “school health service*” OR “school health care*”)

### Results 959 records

| **#** | **Query** | **Limiters** | **Results** |
| --- | --- | --- | --- |
| S6 | S1 AND S2 AND S3 AND S4 | Limiters –  Academic journals | 959 |
| S5 | S1 AND S2 AND S3 AND S4 | Search modes - Boolean/Phrase | 993 |
| S4 | (MM "Healthcare Disparities") OR (MM "Health Care Delivery, Integrated") OR (MH "Health Care Delivery") OR (MH "Health Services Research") OR (MM "Policy Studies") OR (MM "Methodological Research") OR (MM "Usability Study") OR (MM "Quality of Care Research") OR (MH "Outcomes Research") OR (MH "Health Services") OR (MH "Patient Care") OR (MH "School Health Services") OR (MM "School Health Education") OR (MM "School Health Nursing") OR (MM "School Mental Health Services") OR TI( “health care” OR “health intervention*” OR “health care setting*” OR “health setting*” OR “health service*” OR “health sector*” OR “school health service*” OR “school health care*”) OR AB( “health care” OR “health intervention*” OR “health care setting*” OR “health setting*” OR “health service*” OR “health sector*” OR “school health service*” OR “school health care*”) OR SU( “health care” OR “health intervention*” OR “health care setting*” OR “health setting*” OR “health service*” OR “health sector*” OR “school health service*” OR “school health care*”) | Search modes - Boolean/Phrase | 847,549 |
| S3 | TI(“knowledge translation” OR “adopt*” OR “scaling up” OR “scaling out” OR Matching OR linking OR selecting OR tailoring OR developing OR development OR uptake OR unfolding OR translating) OR AB(“knowledge translation” OR “adopt*” OR “scaling up” OR “scaling out” OR Matching OR linking OR selecting OR tailoring OR developing OR development OR uptake OR unfolding OR translating) OR SU(“knowledge translation” OR “adopt*” OR “scaling up” OR “scaling out” OR Matching OR linking OR selecting OR tailoring OR developing OR development OR uptake OR unfolding OR translating) | Search modes - Boolean/Phrase | 914,884 |
| S2 | TI(determinant* OR barrier* OR “hindering factor*” OR “implementation factor*” OR enabler* OR facilitat*) OR AB(determinant* OR barrier* OR “hindering factor*” OR “implementation factor*” OR enabler* OR facilitat*) OR SU(determinant* OR barrier* OR “hindering factor*” OR “implementation factor*” OR enabler* OR facilitat*) | Search modes - Boolean/Phrase | 298,254 |
| S1 | (MH "Implementation Science") OR TI(“implementation science*” OR "implementation strateg*" OR "implementation intervention*" OR "implementation action*" OR "implementation plan*" OR "implementation techni*" OR “health plan implementation” OR “implementation research” OR “implementation scientific research” OR “health care policy”) OR AB(“implementation science*” OR "implementation strateg*" OR "implementation intervention*" OR "implementation action*" OR "implementation plan*" OR "implementation techni*" OR “health plan implementation” OR “implementation research” OR “implementation scientific research” OR “health care policy”) OR SU(“implementation science*” OR "implementation strateg*" OR "implementation intervention*" OR "implementation action*" OR "implementation plan*" OR "implementation techni*" OR “health plan implementation” OR “implementation research” OR “implementation scientific research” OR “health care policy”) | Search modes - Boolean/Phrase | 8,875 |

## Web of Science (Clarivate)

### Search strings

**# Implementation strategy**

TS=("Implementation Science" OR "Health Plan Implementation" OR "implementation strateg*" OR "implementation intervention*" OR "implementation action*" OR "implementation plan*" OR "implementation techni*" OR “health plan implementation” OR “implementation research” OR “implementation scientific research” OR “health care policy”)

**# Determinants**

TS=("Decision Theory" OR determinant* OR barrier* OR “hindering factor*” OR “implementation factor*” OR enabler* OR facilitat*)

**# Matching**

TS= (“knowledge transfer” OR “knowledge translation” OR “adopt*” OR “scaling up” OR “scaling out” OR Matching OR linking OR selecting OR tailoring OR developing OR development OR uptake OR unfolding OR translating)

**# Setting**

TS=("Delivery of Health Care" OR "Health Services Research" OR "Health Care Sector" OR "Patient Care" OR “health care” OR “health intervention*” OR “health care setting*” OR “health setting*” OR “health service*” OR “health sector*” OR “school health service*” OR “school health care*”)

### Results 1.798 records

| **Nr.** | Query | Results |
| --- | --- | --- |
| **#6** | #1 AND #2 AND #3 AND #4 and Article or Review Article or Early Access or Note (Document Types) | [1,798](https://www-webofscience-com.vu-nl.idm.oclc.org/wos/woscc/summary/3884cca3-b118-4c9d-886d-1a1ff8a6747a-64992ef8/relevance/1) |
| **#5** | #1 AND #2 AND #3 AND #4 | [1,843](https://www-webofscience-com.vu-nl.idm.oclc.org/wos/woscc/summary/aad0b63a-4288-484a-8006-7c412d24acf4-64991f93/relevance/1) |
| **#4** | TS=("Delivery of Health Care" OR "Health Services Research" OR "Health Care Sector" OR "Patient Care" OR “health care” OR “health intervention*” OR “health care setting*” OR “health setting*” OR “health service*” OR “health sector*” OR “school health service*” OR “school health care*”) | [648,858](https://www-webofscience-com.vu-nl.idm.oclc.org/wos/woscc/summary/85eeeebd-9669-4996-8c4a-66b525da32de-64991be8/relevance/1) |
| **#3** | TS= (“knowledge transfer” OR “knowledge translation” OR “adopt*” OR “scaling up” OR “scaling out” OR Matching OR linking OR selecting OR tailoring OR developing OR development OR uptake OR unfolding OR translating) | [13,307,580](https://www-webofscience-com.vu-nl.idm.oclc.org/wos/woscc/summary/0b9b4223-8e92-4499-9a32-35d165443e31-64991710/relevance/1) |
| **#2** | TS=("Decision Theory" OR determinant* OR barrier* OR “hindering factor*” OR “implementation factor*” OR enabler* OR facilitat*) | [2,178,925](https://www-webofscience-com.vu-nl.idm.oclc.org/wos/woscc/summary/a5242be1-f015-441d-857d-74e809565b30-64986039/relevance/1) |
| **#1** | TS=("Implementation Science" OR "Health Plan Implementation" OR "implementation strateg*" OR "implementation intervention*" OR "implementation action*" OR "implementation plan*" OR "implementation techni*" OR “health plan implementation” OR “implementation research” OR “implementation scientific research” OR “health care policy”) | [21,486](https://www-webofscience-com.vu-nl.idm.oclc.org/wos/woscc/summary/c74ba0c0-1280-44bc-81d0-103ce513f610-64985abd/relevance/1) |

## Sources for Dutch grey literature

| **Source** | **Organisation type** |
| --- | --- |
| Implementatie bij Zorg voor Innoveren | Network |
| Trimbos – Center for implementation | Applied knowledge centre; intermediary |
| ZonMw implementation portal | Medical research funder |
| Dutch Youth Institute (NJI) | Applied knowledge centre; intermediary |
| Dutch Healthcare institute (ZIN) | Regulator |
| Vilans | Applied knowledge centre; intermediary |
| Institute for responsible medicine use (IVM) | Applied knowledge centre; intermediary; professional association |
| Verenso Dutch Association of Elderly Care Physicians | Applied knowledge centre; professional association |
| Netherlands Institute for Health Services Research (NIVEL) | Applied research institute; intermediary |

The sources were searched for the presence of documents using the following keywords:

- implementation strategy or activity (in Dutch: *implementatiestrategie en -activiteit*)
- matching determinants or barriers and facilitators (in Dutch: *koppelen aan determinanten* or *bevorderende en belemmerende factoren*),
- selecting implementation strategies (in Dutch: *selecteren implementatiestrategieen*)
- implementation methods (in Dutch: *implementatiemethoden*).
