# Supplementary material for: Applied methods for matching implementation strategies to determinants: a scoping review of scientific and grey literature, and qualitative exploration of practice experiences
Source: Implement Sci. 2025 Dec 18;21:14. doi: 10.1186/s13012-025-01477-w (PMC12911165; doi:10.1186/s13012-025-01477-w)
Supplement: Supplementary file 2 — Supplementary Material 2. [file 13012_2025_1477_MOESM2_ESM.docx]

Interview guide Linken Strategieën-Determinanten

***English translation on page 8***

Via 10-15 interviews wordt inhoudelijke informatie bij ervaren implementatie ondersteuners in de (publieke) zorg opgehaald. Deze informatie is aanvullend op en contrasterend van het literatuuronderzoek en richt zich op het verkrijgen van een beter beeld op de wijze waarop in de praktijk implementatie strategieën worden ontwikkeld en met name, de wijze waarop deze worden gekoppeld aan determinanten (dwz. bevorderende en belemmerende factoren).

De interviews worden semigestructureerd afgenomen om te zorgen dat alle relevante onderwerpen aan bod komen terwijl er ook ruimte blijft om door te vragen en bredere input vanuit de gesprekspartners op te halen. De interviews vinden in december 2022 en januari 2023 plaats en worden door leden van de werkgroep uitgevoerd in het Nederlands.

Vanwege de efficiëntie worden de interviews online uitgevoerd. De interviews zullen ongeveer een uur in beslag nemen. Gespreksnotities worden gestructureerd volgens het onderstaande format en niet gedeeld met de deelnemers.

Afspraken voor interviews worden bevestigd met een e-mail waarin ook de gespreksonderwerpen voor het interview staan aangegeven. Onder staat een concepttekst die in de uitnodiging gebruikt kan worden.

*Respondenten*

Op basis van de bestaande netwerken van leden van de werkgroep zijn de volgende organisaties en personen geïdentificeerd. In het identificeren van deze personen zijn geen harde criteria gehanteerd anders dan dat ze ervaring moeten hebben in het aansturen, coördineren, en/of uitvoeren van implementatiewerkzaamheden. Hier kan ook onder worden verstaan verandermanagement, kwaliteitskunde, kennistranslatie, communicatie en scholing, onderzoek, etc. E.e.a. dient gericht te zijn op het implementeren van een nieuwe interventie of manier van werken in een bestaande (publieke) zorgomgeving.

# Email uitnodiging – concepttekst

Beste […],

Graag nodig ik u uit voor deelname aan een interview over de ontwikkeling van implementatie strategieën in de zorgpraktijk. Dit interview maakt deel uit van een onderzoek naar het koppelen van implementatiestrategieën aan determinanten (belemmerende en bevorderende factoren) die deze determinanten effectief adresseren, ook wel het matchen genoemd. Dit onderzoek wordt gefinancierd door en uitgevoerd in opdracht van ZonMw.

**Achtergrond onderzoek**

Er is veel kennis en kunde over implementeren, zowel in de wetenschappelijk literatuur als in de praktijk. Veel implementatieondersteuners, veranderkundigen, zorg- en praktijkprofessionals zijn goed in het identificeren van relevante determinanten (belemmerende en bevorderende factoren) voor het implementeren van een innovatie in de praktijk. Daarnaast is er een veelheid aan implementatiestrategieën bekend en beschikbaar in de literatuur. Echter, het koppelen van geïdentificeerde determinanten aan implementatiestrategieën die deze determinanten effectief kunnen adresseren, ook wel het matchen genoemd, is een complexe aangelegenheid. Vanuit de praktijk en uit wetenschappelijk onderzoek blijkt dat er een behoefte is naar overzicht en kennis van methoden voor het koppelen van determinanten aan strategieën.

Deze kennissynthese bestaat uit een systematisch literatuuronderzoek die we willen complementeren en contrasteren met ervaringen uit de praktijk. Hiervoor bevragen we verschillende mensen die te maken hebben met het implementeren van zorginnovaties naar ervaringen met het ontwikkelen en koppelen van implementatiestrategieën aan determinanten.

**Uw input is waardevol**

Gezien uw ervaring, kennis en kunde op dit onderwerp zouden we graag met u daarover spreken en u uitnodigen voor een semigestructureerd interview. Het zal niet meer dan een uur van uw tijd vragen en u helpt ons en ZonMw inzicht te krijgen in de manier waarop implementatie strategieën in de zorgpraktijk worden ontwikkeld en welke kennis uit de literatuur hierbij wordt toegepast. Dit inzicht is van essentieel belang om ZonMw aanbevelingen te kunnen doen voor de verdere verspreiding en toepassing van kennis over de ontwikkeling van implementatie-strategieën. Uw gegevens zullen vertrouwelijk, geanonimiseerd en op een geaggregeerde manier worden gebruikt. Deelname is geheel vrijwillig en u kunt te allen tijde zonder opgaaf van reden stoppen.

Graag hoor ik van u of u hieraan wilt meewerken. Natuurlijk ben ik beschikbaar om eventuele vragen te beantwoorden.

Mede namens de werkgroep, dr. Erwin Ista, dr. Leti van Bodegom-Vos, dr. Femke van Nassau, en dr. Christiaan Vis, met vriendelijke groet,

[...]

# Gespreksonderwerpen - notitieformat

**Introductie – welkom**

Wie ben je en wat doe je

|  |
| --- |

**Demografie**

| Variabel | Antwoord categorieen | Respons |
| --- | --- | --- |
| Gender | M / F |  |
| Leeftijd | Jaren |  |
| Formele educatie (niveau) | 1 = MBO  2 = HBO / Bachelor  3 = WO / Master  4 = Postdoctoraal |  |
| Jaren professionele ervaring (met implementatie; implementatie gerelateerd) | 1 = 0-5 jaar  2 = 5-10 jaar  3 = 10-15 jaar  4 = > 15 jaar |  |
| (hoofd)functie / rol | 1 = Projectmanager  2 = Teamleider  3 = Veranderkundige  4 = Implementatie-ondersteuner/deskundige  5 = Onderzoeker  Anders: |  |
| Organisatie type (vertegenwoordigd in dit interview) | 1 = Zorginstelling  2 = Kennisinstelling  3 = Netwerk/intermediair  4 = Onderwijsinstelling  5 = Consultancy  Anders: |  |
| Recente (korter dan 5 jaar) nascholing implementatie/cursus gevolgd? | Ja/nee |  |

**Ervaring met implementatie**

Algemeen gesprek over wat hij/zij op dit gebied heeft gedaan inclusief

|  |
| --- |

Hoe verkrijg je ‘implementatiekennis en -vaardigheden’ en hoe houd je die op peil (bv cursus/opleiding; deelname netwerk; praktijkervaring, etc.)

|  |
| --- |

Wat zijn de belangrijkste lessen die je tot dusver hebt geleerd? Voorbeelden van best practices?

|  |
| --- |

**Implementatieproces**

Wat doe je normaal gesproken als je een nieuwe interventie gaat implementeren? Welke stappen doorloop je en wat zijn de belangrijkste acties? Hoe breng je bijvoorbeeld doelen/problemen/determinanten/factoren etc. in kaart?

|  |
| --- |

Kun je een concreet voorbeeld geven?

|  |
| --- |

**Ontwikkelen strategieën**

Hoe ontwikkel je implementatiestrategieën? Welke stappen/acties onderneem je daarin?

|  |
| --- |

Kun je een concreet voorbeeld geven?

|  |
| --- |

Gebruik je daar bijvoorbeeld bestaande strategieën bij en hoe maak je daar keuzes in? Dwz. op basis waarvan bepaal je de strategieën? Gebruik je daar bv een overzicht van mogelijkheden of taxonomie bij en zo ja welke/waarom die? Gebruik van theorieën? Pas je de strategieën ook aan en zo ja hoe/waarom?

|  |
| --- |

Kun je een concreet voorbeeld geven?

|  |
| --- |

De methoden of aanpak die je hebt gebruikt; hoe kijk je daarop terug? Werkt het voor jou? Zou je het nog een keer zo doen of aan andere adviseren?

|  |
| --- |

**Stakeholders**

Welke rol spelen stakeholders hierin en op welke wijze betrek je die daarbij? Hoe bepaal je de stakeholders?

|  |
| --- |

Kun je een concreet voorbeeld geven?

|  |
| --- |

**Praktijk versus onderzoek**

Hoe kijk je tegen de rol van implementatieonderzoek aan in jouw werk?

|  |
| --- |

**Aanbevelingen**

Wat is volgens jou nodig om effectiever en doeltreffender te zijn bij het ontwikkelen of afstemmen van implementatiestrategieën op determinanten, in termen van bijvoorbeeld kennis, vaardigheden, competenties, zelfredzaamheid, enz.

|  |
| --- |

**Afsluiting**

Is alles besproken?

|  |
| --- |

Interview Guide: Linking Strategies-Determinants

Through 10–15 interviews, in-depth information will be collected from experienced implementation support professionals in (public) healthcare. This information complements and contrasts with the literature review and aims to provide a better understanding of how implementation strategies are developed in practice — particularly how these are linked to determinants (i.e., facilitating and hindering factors).

The interviews will be semi-structured to ensure all relevant topics are covered, while still allowing flexibility to explore issues in more depth and gather broader input from participants. The interviews will take place in December 2022 and January 2023 and will be conducted in Dutch by members of the working group.

For efficiency, interviews will be conducted online and will take approximately one hour. Notes from the interviews will be structured according to the format below and will not be shared with participants.

Appointments for interviews will be confirmed by email, which will also outline the interview topics. A draft text for this invitation is provided below.

*Respondents*

Based on existing networks of working group members, the following organizations and individuals have been identified. No strict selection criteria were applied other than that participants must have experience in managing, coordinating, and/or carrying out implementation work. This may include change management, quality improvement, knowledge translation, communication and training, research, etc. The work must involve implementing a new intervention or working method within an existing (public) healthcare setting.

# Invitation Email – Draft Text

Subject: Invitation to participate in an interview on developing implementation strategies in healthcare practice.

Dear […],

I would like to invite you to participate in an interview about the development of implementation strategies in healthcare practice. This interview is part of a study on linking implementation strategies to determinants (barriers and facilitators) that effectively address these determinants — also known as “matching.” This research is funded and commissioned by ZonMw.

### **Background**

There is extensive knowledge and expertise about implementation — both in scientific literature and in practice. Many implementation support professionals, change experts, healthcare, and practice professionals are skilled in identifying relevant determinants (barriers and facilitators) for implementing innovations in practice. Additionally, a wide variety of implementation strategies is known and available in the literature. However, linking identified determinants to implementation strategies that effectively address them (“matching”) is complex.

Both research and practice indicate a need for greater clarity and understanding of methods for linking determinants to strategies.

This knowledge synthesis includes a systematic literature review, which we aim to complement and contrast with real-world experiences. Therefore, we are interviewing professionals involved in implementing healthcare innovations to learn from their experiences in developing and linking implementation strategies to determinants.

### **Your input is valuable**

Given your experience and expertise in this field, we would like to speak with you in a semi-structured interview. The interview will take no more than one hour. Your contribution will help us and ZonMw gain insight into how implementation strategies are developed in healthcare practice and how knowledge from the literature is applied. This insight is essential to provide ZonMw with recommendations for further dissemination and application of knowledge about developing implementation strategies.

Your data will be treated confidentially, anonymized, and reported in aggregate form. Participation is entirely voluntary, and you may withdraw at any time without giving a reason.

Please let me know if you are willing to participate. I am, of course, available to answer any questions.

On behalf of the working group:
Dr. Erwin Ista, Dr. Leti van Bodegom-Vos, Dr. Femke van Nassau, and Dr. Christiaan Vis

Kind regards,
[…]

# Interview Topics – Note Format

**Introduction – Welcome**
Who are you, and what do you do?

|  |
| --- |

### **Demographics**

| Variable | Response categories |
| --- | --- |
| Gender | M / F |
| Age | Years |
| Formal education level | 1 = Vocational (MBO); 2 = Bachelor (HBO); 3 = Master (WO); 4 = Postdoctoral |
| Years of professional experience (implementation-related) | 1 = 0–5 years; 2 = 5–10 years; 3 = 10–15 years; 4 = >15 years |
| Main function / role | 1 = Project manager; 2 = Team leader; 3 = Change expert; 4 = Implementation supporter/specialist; 5 = Researcher; Other: … |
| Organization type | 1 = Healthcare institution; 2 = Knowledge institute; 3 = Network/intermediary; 4 = Educational institution; 5 = Consultancy; Other: … |
| Recent training or course in implementation (< 5 years)? | Yes / No |

### **Experience with Implementation**

- General discussion of the respondent’s activities in this area

|  |
| --- |

- How do you acquire and maintain your implementation knowledge and skills (e.g., training, networks, practical experience)?

|  |
| --- |

- What are the most important lessons you have learned so far? Examples of best practices?

|  |
| --- |

### **Implementation Process**

- What steps do you normally take when implementing a new intervention? How do you identify goals, problems, determinants, or factors?

|  |
| --- |

Can you give a concrete example?

|  |
| --- |

### **Developing Strategies**

- How do you develop implementation strategies? What actions or steps do you take?

|  |
| --- |

- Do you use existing strategies, and how do you choose among them?

|  |
| --- |

On what basis do you select them? Do you use an overview or taxonomy? Which one and why? Do you use theories? Do you adapt the strategies? How and why?

|  |
| --- |

Can you give a concrete example?

|  |
| --- |

How do you reflect on the methods or approaches you used? Did they work? Would you recommend them?

|  |
| --- |

### **Stakeholders**

What role do stakeholders play? How do you identify and involve them?

|  |
| --- |

Can you provide an example?

|  |
| --- |

### **Practice vs. Research**

How do you view the role of implementation research in your work?

|  |
| --- |

### **Recommendations**

What, in your view, is needed to be more effective and efficient in developing or matching implementation strategies to determinants (in terms of knowledge, skills, competencies, autonomy, etc.)?

|  |
| --- |

### **Closing**

Has everything been discussed?

|  |
| --- |
