# Supplementary material for: Applied methods for matching implementation strategies to determinants: a scoping review of scientific and grey literature, and qualitative exploration of practice experiences
Source: Implement Sci. 2025 Dec 18;21:14. doi: 10.1186/s13012-025-01477-w (PMC12911165; doi:10.1186/s13012-025-01477-w)
Supplement: Supplementary file 3 — Supplementary Material 3. [file 13012_2025_1477_MOESM3_ESM.docx]

Appendix 3: General information about the included studies, the underpinning, steps and core elements of the methods for matching implementation strategies

| **Author (year)** | **Setting** | **Target population** | **Intervention type** | **Underpinning** | | | **Steps** | **Core elements** |
| --- | --- | --- | --- | --- | --- | --- | --- | --- |
|  |  |  |  | **Determinants** | **Matching** | **Stakeholders** |  |  |
| Aakhus (2015) | Primary care | Elderly | Guideline | TICD-checklist | Logic modelling | Brainstorming; focus groups | 4 | Brainstorming with stakeholders, followed by prioritising the strategies, taking into account the perceived impact, evidence base, feasibility and cost of the strategies. A five-step framework was used to analyse the results, including a standardised procedure to rank and select strategies based on feasibility and utility. |
| Baillie (2017) | Primary care | Healthy adults at risk | Guideline | Survey data | Logic modelling; driver diagram | Survey data; interviews | 4 | Iterative process in which survey data are used to identify determinants that are characteristics of the health system or personnel involved. Development of causal paths of determinants using the driver diagram. Alignment and refinement of causal pathways and strategies takes place with by stakeholders. |
| Baldwin (2022) | Primary care | Clinicians | Procedural | CFIR | ERIC; op basis van consensus | Combined group activity | 3 | Combined group exercise with experts and stakeholders developing a matrix of strategies and determinants. When both 30% of experts and stakeholders indicated agreement, the strategy was selected and examples were developed using CFIR and ERIC frameworks |
| Becker-Haimes (2022) | Primary care | Health professionals | Procedural | Literature review; BCW | ERIC; BCW | Participatory research; interviews; observations | 3 | Hypothesis-based; identified behavioural barriers were linked to key principles from behavioural science. The hypothesised determinants were validated through expert consultation, literature review and key stakeholders. Based on these results, implementation strategies were developed. |
| Boehmer (2019) | Community; public health | Patients | Procedural | Experience-based | AIDED Toolkit | Interviews | 2 | Lessons from previous projects were used to identify problems in the clinical process. The team proposed solutions to these problems, leading to an implementation toolkit. The toolkit was further designed and validated with stakeholder support. |
| Brons (2022) | Primary care | Youth | Behavioural (lifestyle)^6^ | Int.M | Int.M | Group discussions met stakeholders | 6 | Includes needs assessment and specification of objectives, followed by selection of theory-based interventions, development of a programme and implementation plan, and programme evaluation. |
| Chanfreau-Coffinier (2019) | Health care organisations | Policy makers; health professionals | Behavioural (clinical) | CFIR | ERIC; logic modelling | Interviews | 2 | Based on qualitative statements from experts and participants, determinants were identified using CFIR and implementation strategies selected using ERIC. A logic model was then developed with planned work (inputs and activities) and intended results (outputs, outcomes and impacts) for programme implementation. |
| Danila (2016) | Primary care | Patients | Behavioural (lifestyle) | Brainstorming | Surveys | Brainstorming; Surveys | 4 | Brainstorming of potential determinants with stakeholders, followed by selection of adaptable determinants through expert advice. Prioritisation of the resulting determinants is done through Surveys among participants. The implementation strategy is then tailored to race/ethnicity and the selected determinants |
| Fernandez (2019) | Community; specialised | N.A. | Combination | Imp.M | Imp.M | Various methods | 6 | First, behavioural and environmental outcomes are identified. Then, goals for change in the determinants of those behavioural and contextual causes and the objectives of the intervention programme are specified. Next, theoretical and evidence-based behaviour change methods that influence the determinants are identified and translated into practical applications that fit the context. |
| French (2012) | Primary care | Patients | Behavioural (clinical) | TDF | TDF | Various methods incl. interviews, focus groups, etc. | 4 | Guiding questions guide the choice of components of an implementation intervention: identifying the problem (who should do what differently), assessing the problem (using a theoretical framework, which determinants should be addressed), forming possible solutions (which intervention components could address the determinants), and evaluating the chosen intervention. Literature, theories and stakeholder views are consulted in the different steps. |
| Fry (2020) | Specialised; hospital care | Health professionals | Behavioural (clinical) | Experience-based; ACT; TDF | BCW | Surveys | 2 | Previous findings on determinants were categorised and mapped using the TDF. BCW was then used to identify targeted behaviour change strategies to strengthen incentives and reduce barriers. |
| Grandes (2017) | Primary care | Community workers | Behavioural (lifestyle) | Int.M | Plan-do-study-act | Brainstorming | 4 | Bottom-up process of dialogue, discussion and consensus between a multi-professional front-line team and community members for joint decision-making on actions to be included in the strategy. The IM was used as a guide to structure the brainstorming and included three steps: descriptive, creative, pilot. The Plan-Do-Study-Act approach was used to structure the strategy. |
| Hetrick (2018) | Specialised; hospital care | Patients; health professionals | Behavioural (lifestyle) | TDF | Literature review; TDF | Interviews | 3 | Implementation strategies were developed using interviews with clinicians to identify determinants, empirical evidence on the effects of implementation interventions, and understanding of the active elements of implementation strategies and theorising about pathways to change. TDF was used to structure the process. |
| Highfield (2018) | Primary care | Patients | Behavioural (lifestyle) | CFIR | Int.M step 5; Social-cognitive theory, Diffusion of Innovation | Brainstorming | 4 | IM step 5 was used to guide implementation strategy planning. CFIR was used to identify determinants. A planning committee conducted a stakeholder analysis, which was then involved in identifying determinants in brainstorming sessions. The planning committee developed change objectives and matrices. Strategies were developed using the change matrices and the principles of Social Cognitive Theory, and the Diffusion of Innovation to define the change components of the intervention. |
| Howell (2022) | Specialised; hospital care | Clinicians | Behavioural (clinical) | NPT; CFIR | CFIR-ERIC | Surveys; interviews; focus groups | 3 | Multiple methods (surveys, interviews, focus groups) were used to collect information on determinants from stakeholders. These used the NPT and other (existing) questionnaires. Determinants were mapped using CFIR and then the CFIR-ERIC Strategy Matching tool was used to select implementation strategies that could then be matched to local needs. |
| Ibekwe (2022) | Community; public health | Community workers | Behavioural (lifestyle) | Imp.M | Imp.M matrices of change | Brainstorming | 5 | Implementation Mapping was used, which consists of 5 steps: a needs assessment, creating change matrices, choosing a theoretical basis and designing the strategy, creating an implementation plan, and evaluating the results of that plan. This can be done for three phases of implementation: adoption, implementation and maintenance. Matrices with performance objectives linked to behavioural determinants provide information on change objectives. Change objectives are then used to create a theory-based plan for each implementation phase. |
| Jäger (2014) | Primary care | Clinicians; patients, carers | Behavioural (clinical) | Implementation Fidelity framework | Implementation Fidelity framework | Interviews | 2 | Various methods (group interviews with stakeholders) were used to identify strategies to overcome the identified barriers. The identified strategies were prioritised based on criteria for feasibility and assumed impact. |
| Kansal (2022) | Specialised; hospital care | Patients | Behavioural (clinical) | Interviews | Interview; CFIR-ERIC | Interviews | 2 | Determinants (or groups of) were identified using interviews and qualitative thematic analysis. Determinants were then linked to suggested strategies from the interview. Barriers without suggestion were linked to strategies using the CFIR-ERIC matrix. |
| Kernan (2023) | Community; public health | Clinicians; healthy elderly at risk | Behavioural (lifestyle) | Experience-based | CFIR-ERIC; eDelphi; consensus-based | Interviews; eDelphi | 3 | The first step involved collecting existing experiences and strategies used and information on observed determinants. In the second step, the CFIR-ERIC Matching Tool is used to identify implementation strategies to address the identified determinants. In the third step, an e-Delphi method was used to support the prioritisation of implementation strategies at the patient, professional and organisational levels. |
| Kirk (2022) | Specialised; hospital care | Patients | Behavioural (clinical) | Conjoint analysis | ERIC; consensus based | Co-design workshops | 3 | Possible implementation strategies were extracted from the ERIC compilation. Based on this compilation, examples were written to illustrate application and relevance. The selection and alignment of implementation strategies to address barriers and determine the outcome goals of the implementation strategies was based on a joint analysis of identified and prioritised determinants. |
| Kurlander (2022) | Specialised; hospital care | Patients | Behavioural (clinical) | Imp.M | Imp.M | Participatory planning group; Interviews | 2 | Implementation Mapping was used, which consists of 5 steps: a needs assessment, creating change matrices, choosing a theoretical basis and designing the strategy, creating an implementation plan, and evaluating the results of that plan. This can be done for three phases of implementation: adoption, implementation and maintenance. |
| Kwok (2020) | Pre-school | Healthy youth at risk | Behavioural (school) | Concept mapping | TDF; BCT | Concept-mapping | 5 | Concept mapping offers an approach that involves stakeholders in a series of sequential tasks. These include: (1) Brainstorming and statement analysis, (2) structuring statements (sorting and reviewing) by stakeholders, (3) concept mapping analysis, and (4) data interpretation, (5) verifying the working mechanisms of the prioritised list of implementation strategies using TDF and behaviour change literature. |
| Li (2021) | Specialised; hospital care | Clinicians | Procedural | Surveys; interviews | CFIR-ERIC | Surveys; interviews | 2 | Contextual determinants were identified in consultation with various stakeholders through both Surveys and interviews. After conducting context assessments, identifying barriers and facilitators, and soliciting stakeholder input on strategies, the CFIR-ERIC tool was used to help select and align the strategy to address the identified determinants. |
| McArthur (2018) | Rehabilitation | Clinicians | Behavioural (clinical) | KTA | BCW | Interviews; focus groups | 3 | Determinants were assessed according to the knowledge use and intervention selection steps of the KTA cycle. The BCW was used as a framework for behavioural analysis and strategy element selection. This involved defining the behavioural problem, specifying the target behaviour, identifying the needs for change (its determinants), and identifying the required strategy components. |
| Midboe (2018) | Primary care | Patients | Behavioural (clinical) | CFIR | CFIR; RE-AIM | Team-based | 2 | Two external and internal implementation development and facilitation teams were established. These teams focused on identifying determinants and appropriate implementation strategies using CFIR. RE-AIM was used to monitor implementation progress. |
| Mills (2015) | Community; public health | Patients | Guideline | Concept-mapping; chronic care model | Concept-mapping; chronic care model | Concept-mapping (brainstorming; group discussion) | 3 | Concept mapping involved (1) Brainstorming, (2) sorting and reviewing, and (3) interpretation of the resulting concept map. Participants generated a large number of ideas for determinants and strategies that were sorted into clusters and grouped within a partnership framework at system, community and individual levels. |
| Moise (2020) | Primary care | Patients | Behavioural (lifestyle) | BCW; COM-B | BCW; COM-B; APEASE | Interviews | 4 | A multidisciplinary team used mixed-methods to develop, refine, locally adapt and finalise components of the implementation strategy. In line with the BCW, barriers were coded into COM-B categories by members of the research team, followed by mapping the determinants into 9 broad categories through which a strategy can change behaviour. APEASE criteria were used (by the research team) to assess the feasibility of mapped strategies, policy categories and behaviour change techniques. Through interviews, the feasibility and acceptability of the strategies were confirmed from the perspective of different stakeholders in the local clinical context. |
| Moore (2014) | Specialised; hospital care | Elderly patients | Behavioural (clinical) | Experience-based; focus groups | BCT; COM-B | Focus groups | 5 | Five phases were completed: (1) creation of a list of possible implementation activities based on previous experience and data, (2) focus groups with stakeholders to identify unique barriers to behaviour change, (3) collection of data on post-implementation adaptations of the intervention, (4) mapping of barriers and implementation activities to the COM-B system, and (5), development of a manual for adapting and implementing similar interventions. |
| Munir (2013) | Primary care | Patients | Guideline | Literature review; Int.M | Int.M | Focus groups | 6 | Following the Intervention Mapping approach: 1) a needs assessment; 2) the identification of outcomes, performance targets and change objectives; 3) the selection of theory-based methods and practical strategies; 4) the development of programme components and materials; 5) planning for programme adoption, implementation and sustainability; and 6) a plan for evaluation. |
| O'Grady (2022) | Specialised; hospital care | N.A. | Behavioural (clinical) | Int.M | Int.M; i-PARIHS; expert-based | Interviews | 3 | After identifying the determinants the strategy was mapped in four steps: (1) the researchers used data from a previous step to assess implementation needs; (2) the needs were presented to a stakeholder group to set goals. Step (3) involved identifying theory-based, practical, discrete implementation strategies to meet the needs, goals and objectives of step 2. Strategy selection was guided by the following principles: (a) appropriateness for the setting, (b) replicability and generalisability, (c) feasibility, and (d) alignment with the i-PARIHS components in terms of needs, constraints, resources and context. Implementation strategies were drawn from the i-PARIHS framework and other lists of implementation strategies. Step (4) involved designing and operationalising the implementation strategy. The feasibility, acceptability and preliminary effectiveness of the implementation strategy were then assessed. |
| Jolles (2022) | Primary care | Healthy youth at risk | Combination | EPIS; Imp.M | Imp.M | Brainstorming; group discussions | 4 | Strategy development involved four steps: (1) a needs and resource assessment and identification of stakeholders and implementers; (2) formulation of desired implementation outcomes, performance targets and determinants; preparation of change matrices, (3) development of change methodologies and practical applications for using the programme, and (4) preparation of implementation protocols and materials. |
| Piat (2022) | Primary care | Patients | Guideline | Consensus-based | Consensus-based | Focus groups; Brainstorming | 6 | A series of 12 structured meetings with implementation teams consisting of local stakeholders were held to develop an implementation strategy. The process consisted of 6 steps: (1) selecting a clinical practice guideline, (2) defining the target intervention or behaviour, (3) identifying barriers and facilitators, (4) defining and specifying implementation strategies, (5) engaging (other) stakeholders relevant to the implementation strategies, and (6) writing an implementation plan. Key elements of the 12 meetings included the value of consensus building among implementation team members and the resulting shifting balance of power. |
| Podolak (2017) | Community; public health | Healthy adults at risk | Combination | Scenario-based planning; existential phenomenology | Scenario-based planning; existential phenomenology | Participatory action research | 3 | The methodological steps of (1) participatory action research consisting of (a) team building, (b) diagnosis and planning, (c) action and reflection, and (d) specification of the learning process; (2) scenario-based planning consisting of (a) scope delineation, (b) trend and uncertainty analysis, (c) description of phenomena, (d) scenario building, (e) strategy formulation, and finally (3) existential phenomenology consisting of (a) delineation of objectives, (b) joint development of decision-making guidelines with stakeholders and assessment of interaction between participants, (c) development of composite descriptions, and (d) quality assessment. |
| Powell (2017) | Community; public health | Healthy adults at risk | Combination | Concept mapping; group modelling; conjoint analysis; Int.M | Concept mapping; group modelling; conjoint analysis; Int.M | Varies | Varies | This article reviews four methods for selecting and developing implementation strategies: (1) concept mapping, (2) group modelling, (3) conjoint analysis, and (4) Intervention Mapping. All methods involve stakeholders in different ways. Concept mapping is a mixed method of organising ideas from a group into a common framework. Group modelling is characterised by using social systems, especially the patient, to obtain feedback from stakeholders to study causality (variables, relationships and feedback) to identify opportunities for improvement. Conjoint analysis is a quantitative method that involves stakeholders to determine how they value different characteristics of interventions and implementation strategies. Intervention mapping is based on mixed-methods research, theory, stakeholder input and a step-by-step intervention development process, including the development of an implementation plan. |
| Powell (2020) | Community; mental health | Youth | Behavioural (clinical) | Int.M | Int.M | Group discussions; various methods | 5 | Training and education in using Intervention Mapping to develop implementation strategies; The Collaborative Organisational Approach to Selecting and Tailoring Implementation Strategies (COAST-IS) is an intervention designed to increase the effectiveness of implementing and supporting evidence-based practices. COAST-IS enables organisation leaders and clinicians to use Intervention Mapping to select and tailor implementation strategies to their specific needs. Intervention Mapping is a multi-step process that integrates theory, evidence and stakeholder perspectives to ensure that implementation strategies effectively address key determinants of change. |
| Reuter (2022) | Specialised; hospital care | Patients | Behavioural (clinical) | Literature review | BCW; COM-B; APEASE; ERIC | Bi-lateral and group discussions | 10 | The methodology applied included three phases. Phase 1 included a narrative literature review to identify barriers and facilitators. Phase 2 used a multi-step framework to develop an implementation strategy that simultaneously targeted healthcare systems, providers and patients. In phase 3, stakeholders were involved to adapt the implementation strategy to the local context. The multi-step plan consisted of 8 steps: (1-4) understanding the behaviour, (5-6) identifying intervention options, (7-8) identifying behaviour change techniques, content and implementation options. BCW, COM-B and the APEASE criteria were used in these steps. ERIC and several stakeholder meetings were used to adapt the strategy to the local context in phase 3. |
| Riordan (2020) | Primary care | Patients | Behavioural (clinical) | TDF; BCW | TDF; BCW; APEASE | Interviews; group discussions | 5 | In steps 1 to 3, determinants and needs were identified. In step 4, stakeholders were consulted to check the ‘fit’ of the strategy with existing processes. In step 5, the APEASE criteria were applied to select the final components of the implementation strategy. |
| Riphagen-Dalhuisen (2013) | Specialised; hospital care | Health professionals | Behavioural (lifestyle) | Int.M | Int.M | Group discussions; various methods | 6 | Following the Intervention Mapping approach with: 1) needs assessment; 2) identification of proximal programme objectives; 3) development of theory-based methods and practical strategies; 4) programme planning; 5) programme approval and implementation; and 6) programme evaluation. |
| Robitaille (2019) | N.A. | Patients | Guideline | KTA | KTA | Survey | 6 | The action cycle of KTA was applied: (1) identification of the problem, (2) identification, evaluation and selection of knowledge relevant to the problem, (3) adaptation of the identified knowledge to the local context, (4) assessment of barriers and facilitators to knowledge use, (5) selection, targeting and implementation of the interventions to promote knowledge use (through education) and (6) monitoring of knowledge use. |
| Rogal (2020) | Specialised; hospital care | Patients | Guideline | Literature review | ERIC; GTO | Interviews; group discussions; survey data | 10 | GTO is stakeholder-driven, col- laborative and easy to understand. GTO is organised as 10 self-explanatory steps (e.g. goal setting, planning, evaluation, quality improvement) and uses strategies such as facilitation, written aids that structure decision-making in the 10 steps, and monitoring and feedback of evaluation data. GTO is evidence-based. |
| Ross (2018) | Primary care | Patients | Behavioural (clinical) | Literature review; Grol & Wensing’s model of implementation | EPOC; NPT | Interviews | 9 | Strategy development involved 9 steps: (1) synthesis of evidence; (2) stakeholder engagement; (3) setting selection; (4) implementation strategy selection; (5) implementation strategy execution; (6) feedback collection; (7) user interviews; (8) usage data collection; and (9) finalisation of adaptations. Potential strategies (step 4) were selected from the EPOC framework and used evidence collected in the previous phases; evidence synthesis and implementation context assessment. The research team also used NPT to select strategies that would trigger change based on increasing coherence, cognitive participation, collective action and reflexive monitoring. |
| Roth (2021) | Primary care | N.A. | Behavioural (clinical) | Int.M | Int.M; Social cognitive theory; Diffusion of Innovation; ERIC | Interviews | 4 | Strategy development involved steps 1-4 of Intervention Mapping: (1) identification of barriers and facilitators, (2) development of a roadmap to implementation, (3) design of strategy components, and (4) implementation of the implementation strategies. In step 3, the research team identified two theories as most relevant to address the determinants from step 1: the Social cognitive theory and the Diffusion of Innovation. ERIC was used to operationalise the insights from these theories into more specific strategies. |
| Rusch (2021) | Community; public health | N.A. | Combination | Implementation Roadmap; consensus-based | Implementation roadmap; consensus-based | Interviews | 7 | The community-informed Implementation Roadmap approach involves seven tasks: (1) identifying high-priority needs, (2) involving stakeholders, (3) selecting key metrics and key performance indicators, (4) assessing contextual factors affecting intervention use and/or implementation, (5) clarifying key features and (6) refining the strategy, and (7) adapting the strategy to the local context. |
| Shrubsole (2022) | Primary care | Health professionals | Behavioural (clinical) | TDF | TDF; ERIC; BCT | Interviews | 8 | The action cycle of TDF includes eight stages, including (1) identification of the problem, (2) identification, assessment and selection of knowledge relevant to the problem, (3) adaptation of knowledge to the local context, (4) assessment of barriers and facilitators to knowledge use, (5) selection, alignment and implementation of strategies to promote knowledge use, (6) monitoring of knowledge use, (7) evaluation of results, and (8) maintenance of knowledge use. Step 5 involved mapping identified barriers to TDF domains, identification and selection of strategies for BCT behaviour change using TDF and ERIC. |
| Simpson (2013) | N.A. | N.A. | Procedural | Experience-based (researchers) | Experience-based (researchers); Algorithm based | Sense making | 3 | TECH focuses on sensemaking and provides a qualitative group process based on complexity theory to assess and interpret implementation problems and identify and evaluate implementation strategies. Sens-making is done through an open dialogue between core members of the implementation team to generate ideas for solutions. Once potential solution strategies were collected, the research team considered whether the proposed solution(s) would address the challenge, support the integrity of the research design and the ability of the research team to successfully implement the research protocol. An algorithm was used to systematically evaluate the strategies. This algorithm included questions such as: To what extent will the proposed solutions overcome the challenge? Will the new strategy negatively affect the integrity of the study? Is the solution strategy compatible with the research environment? Does the team have the resources to successfully implement the new strategy (e.g. staff, money, time)? What regulatory issues need to be addressed before implementing the new strategy? |
| Sinnema (2015) | Primary care | General practitioners | Behavioural (clinical) | Interviews | Expert opinion-based checklist | Interviews | 3 | This method focused on tailored implementation which consists of prospective and iterative process for to develop and align implementation strategies with local determinants. Determinants were identified by the research team through interviews with local stakeholders (GPs). This resulted in a checklist that served as a basis for the research team to develop an initial list of possible implementation strategies. These were reviewed by experts and fed back to stakeholders. Implementation was monitored periodically and fed back to the experts to review possible adjustments to the strategies. |
| Stewart (2020) | Community; public health | Community workers | Behavioural (clinical) | NUDGE; Behavioural economics | Brainstorming | Brainstorming | 5 | NUDGE is a systematic approach to designing implementation strategies, based on behavioural economics, and innovation methods. The premise is that people rarely behave or make decisions rationally. NUDGE is driven by researchers and consists of 5 steps: (1) Narrow: specify relevant behavioural goal; (2) Understand: assess the context of the behaviour by assessing the decision-making process and related actions; (3) Discover: Identify related barriers to the target behaviour by unifying the contextual research from the previous step with core principles (cognitive biases and heuristic thinking) from behavioural science in a structured brainstorming process around the cues, alternatives and meanings of the target behaviour; (4) Generate: design implementation strategies using the outcomes of the Brainstorming; (5) Evaluate: assess the impact of the implementation strategies through iterative prototyping and piloting. |
| Teachout (2021) | Primary care | Healthy adults at risk | Guideline | Change management; program assessment guide | Program Theory of Change; Program Implementation | Brainstorming; various methods | 4 | This 4-stage framework is based on the theory of change and an adapted version of the operational research prioritisation table from the programme assessment guide. It includes (1) linking the theory of change to programme implementation (development and adaptation of the theory of change; linking implementation strategies to determinants in the change theory model), (2) development of an implementation research agenda (identification of gaps in implementation knowledge and measures; development and prioritisation of implementation research questions), (3) implementation of implementation research (identification of objectives, methods and research design, implementation), (4) analysis of findings and preparation and dissemination of recommendations. |
| Van Noort (2020) | Specialised; hospital care | Patients and nurses | Combination | Int.M | Int.M | Interviews; panel discussion | 6 | Followed the Intervention Mapping approach consisting of six steps: (1) formulation of the logical model of the problem, (2) identification of programme outcomes and objectives, (3) programme design, (4) programme production, (5): developing a programme implementation plan, and (6) developing an evaluation plan. |
| Van Oers (2021) | Primary care | Patients | Behavioural (clinical) | CFIR | CFIR-ERIC | - | 2 | Retrospective description of an implementation. Part A) consisted of a qualitative description of the key determinants of successful intervention implementation. Part B) consisted of an evaluation of current barriers in the implementation process and matching possible future strategies to reduce these barriers using the CFIR-ERIC Implementation Strategy Matching tool and a qualitative description of identified barriers and strategies previously used by experts. |
| Van Sluisveld (2013) | Specialised; hospital care | Patients | Combination | Routine care data; Literature review | Literature review; Int.M | Surveys; interviews | 5 | Intervention Mapping was preceded by a review of routine data on target behaviour (variation in readmissions and mortality after discharge), a systematic review of effective interventions, assessment of adherence, and an evaluation of barriers and facilitators to implementing interventions. An implementation strategy was developed based on the data and effective implementation strategies from the literature using the intervention mapping method. |
| Versluis (2020) | Primary care | Health professionals; researchers | Guideline | CFIR | AACTT | - | 5 | In developing the strategy, the CFIR was used to identify determinants. The AACTT framework was used to develop worksheets for designing the implementation strategy, which consists of the following steps: (1) specify the intervention, (2) define the problem, (3) specify the desired implementation behaviour, and (4) choose and (5) evaluate the implementation strategy. |
| Waltz (2019) | Primary care | GPs and researchers | Combination | CFIR | CFIR-ERIC; expert-based | Surveys | 2 | Expert-driven approach in which experts matched and ranked CFIR constructs of determinants to the ERIC framework of implementation strategies. The article presents the CFIR-ERIC Implementation Strategy Matching Tool. The tool can be used to generate a list of ERIC strategies that implementation supporters or researchers may consider to address one or more determinants from the CFIR framework. The matching is based on expert opinion, while the identification of barriers is done on the spot. Proposed strategies are given a recommendation of 1 or 2 levels, depending on the degree of consensus reached by the experts in matching a particular determinant. Subsequently, the list of strategies can serve as a starting point for operationalising a work plan. |
| Watkins (2017) | Primary care | Community workers; researchers | Behavioural (clinical) | Chronic care model | Chronic care model; literature review and experience-based | Focus groups; interviews | 5 | The Chronic care model provides guidance in translating general ideas for quality improvement into specific applications. The model states that in order to improve health outcomes, the healthcare system must consider six elements of care. In developing the implementation strategy, the following five steps were applied: (1) development of protocols; (2) conduct focus groups/interviews to identify key themes; (3) identify barriers and design implementation strategies; (4) test draft strategies; (5) evaluate pilot results, revise strategy/processes & include in manual. Identified determinants were mapped to the six elements of the chronic care model to structure the analysis. Implementation strategies (step 3) were identified both from a Literature review and through the collective experience of the team. |
| Wensing (2017) | Primary care | Patients | Guideline | TICD-checklist | Consensus-based | (group) interviews; Surveys | 3 | The TICD project applied 3 consecutive phases: (a) an exploration of determinants of practice related to the targeted conditions, using interviews and Surveys with health care providers (see Aakhus 2015), (b) group interviews with various stakeholders to gather suggestions for educational, organisational and other interventions to address determinants of practice, and (c) cluster randomised trials of a tailored implementation programme, based on an understanding of determinants and interventions, and related process evaluations. The latter aimed to establish the effectiveness of tailored implementation. |
| Wilkinson (2022) | Primary care | Health professionals | Behavioural (clinical) | TDF; BCW; COM-B | TDF; BCW; COM-B | Interviews | 2 | The development of implementation strategies consisted of two phases: (1) interviews with practitioners to identify key theme determinants, and (2) determinant mapping in TDF and BCW/COM-B to identify effective strategies. |
| Wilson (2022) | Primary care | Patients | Guideline | Panel-based | Panel-based | Surveys; interviews | 2 | Implementation strategies were developed based on Survey and interview data with stakeholders and experts. The project involved multiple stakeholders from health systems, manufacturers, governments, patient advocacy groups and industry to study the experiences of health systems in using particular interventions. Representatives of these stakeholders formed an expert panel. The iterative work of the expert panel led to the development of an implementation roadmap, as well as barriers and strategies to overcome them. |
| Zhao (2022) | Primary care | Health professionals; policy makers; patients | Combination | SMILE | SMILE; I-PARIHS | - | 3 | The SMILE framework provides guidance on using social media as a knowledge-translation strategy to inform healthcare practices and decision-making. It has six main constructs: (1) developers, (2) messages and delivery strategies, (3) recipients, (4) context, (5) triggers, and (6) outcomes. Different suggestions for implementation strategies are made for each of these constructs. However, SMILE should be considered as a heuristic tool for developing social media interventions to promote evidence use. It should be used in conjunction with process frameworks such as I-PARIHS to guide implementation. SMILE is based on 5 theories including BCT/COM-B, Fogg behaviour model, and Diffusion of Innovation. |

**Articles included in review**

Aakhus, E., Granlund, I., Oxman, A. D., & Flottorp, S. A. (2015). Tailoring interventions to implement recommendations for the treatment of elderly patients with depression: a qualitative study. Int J Ment Health Syst, 9, 36. https://doi.org/10.1186/s13033-015-0027-5

Bailie, J., Matthews, V., Laycock, A., Schultz, R., Burgess, C. P., Peiris, D., Larkins, S., & Bailie, R. (2017). Improving preventive health care in Aboriginal and Torres Strait Islander primary care settings. Global Health, 13(1), 48. https://doi.org/10.1186/s12992-017-0267-z

Baldwin, L. M., Tuzzio, L., Cole, A. M., Holden, E., Powell, J. A., & Parchman, M. L. (2022). Tailoring Implementation Strategies for Cardiovascular Disease Risk Calculator Adoption in Primary Care Clinics. J Am Board Fam Med, 35(6), 1143-1155. https://doi.org/10.3122/jabfm.2022.210449R1

Becker-Haimes, E. M., Ramesh, B., Buck, J. E., Nuske, H. J., Zentgraf, K. A., Stewart, R. E., Buttenheim, A., & Mandell, D. S. (2022). Comparing output from two methods of participatory design for developing implementation strategies: traditional contextual inquiry vs. rapid crowd sourcing. Implement Sci, 17(1), 46. https://doi.org/10.1186/s13012-022-01220-9

Boehmer, K. R., Holland, D. E., & Vanderboom, C. E. (2019). Identifying and addressing gaps in the implementation of a community care team for care of Patients with multiple chronic conditions. BMC Health Serv Res, 19(1), 843. https://doi.org/10.1186/s12913-019-4709-6

Brons, A., Braam, K., Broekema, A., Timmerman, A., Millenaar, K., Engelbert, R., Krose, B., & Visser, B. (2022). Translating Promoting Factors and Behavior Change Principles Into a Blended and Technology-Supported Intervention to Stimulate Physical Activity in Children With Asthma (Foxfit): Design Study. JMIR Form Res, 6(7), e34121. https://doi.org/10.2196/34121

Chanfreau-Coffinier, C., Peredo, J., Russell, M. M., Yano, E. M., Hamilton, A. B., Lerner, B., Provenzale, D., Knight, S. J., Voils, C. I., & Scheuner, M. T. (2019). A logic model for precision medicine implementation informed by stakeholder views and implementation science. Genet Med, 21(5), 1139-1154. https://doi.org/10.1038/s41436-018-0315-y

Danila, M. I., Outman, R. C., Rahn, E. J., Mudano, A. S., Thomas, T. F., Redden, D. T., Allison, J. J., Anderson, F. A., Anderson, J. P., Cram, P. M., Curtis, J. R., Fraenkel, L., Greenspan, S. L., LaCroix, A. Z., Majumdar, S. R., Miller, M. J., Nieves, J. W., Safford, M. M., Silverman, S. L., . . . Saag, K. G. (2016). A multi-modal intervention for Activating Patients at Risk for Osteoporosis (APROPOS): Rationale, design, and uptake of online study intervention material. Contemp Clin Trials Commun, 4, 14-24. https://doi.org/10.1016/j.conctc.2016.06.010

Fernandez, M. E., Ruiter, R. A. C., Markham, C. M., & Kok, G. (2019). Intervention Mapping: Theory- and Evidence-Based Health Promotion Program Planning: Perspective and Examples. Front Public Health, 7, 209. https://doi.org/10.3389/fpubh.2019.00209

French, S. D., Green, S. E., O'Connor, D. A., McKenzie, J. E., Francis, J. J., Michie, S., Buchbinder, R., Schattner, P., Spike, N., & Grimshaw, J. M. (2012). Developing theory-informed behaviour change interventions to implement evidence into practice: a systematic approach using the Theoretical Domains Framework. Implement Sci, 7, 38. https://doi.org/10.1186/1748-5908-7-38

Fry, M., Elliott, R., Fitzpatrick, L., Warton, J., & Curtis, K. (2020). Measuring nurses' perceptions of their work environment and linking with behaviour change theories and implementation strategies to support evidence based practice change. Appl Nurs Res, 56, 151374. https://doi.org/10.1016/j.apnr.2020.151374

Grandes, G., Sanchez, A., Cortada, J. M., Pombo, H., Martinez, C., Balague, L., Corrales, M. H., de la Pena, E., Mugica, J., Gorostiza, E., & group, P. V. S. (2017). Collaborative modeling of an implementation strategy: a case study to integrate health promotion in primary and community care. BMC Res Notes, 10(1), 699. https://doi.org/10.1186/s13104-017-3040-8

Hetrick, S. E., O'Connor, D. A., Stavely, H., Hughes, F., Pennell, K., Killackey, E., & McGorry, P. D. (2018). Development of an implementation guide to facilitate the roll-out of early intervention services for psychosis. Early Interv Psychiatry, 12(6), 1100-1111. https://doi.org/10.1111/eip.12420

Highfield, L., Valerio, M. A., Fernandez, M. E., & Eldridge-Bartholomew, L. K. (2018). Development of an Implementation Intervention Using Intervention Mapping to Increase Mammography Among Low Income Women. Front Public Health, 6, 300. https://doi.org/10.3389/fpubh.2018.00300

Howell, D., Powis, M., Kirkby, R., Amernic, H., Moody, L., Bryant-Lukosius, D., O'Brien, M. A., Rask, S., & Krzyzanowska, M. (2022). Improving the quality of self-management support in ambulatory cancer care: a mixed-method study of organisational and clinician readiness, barriers and enablers for tailoring of implementation strategies to multisites. BMJ Qual Saf, 31(1), 12-22. https://doi.org/10.1136/bmjqs-2020-012051

Ibekwe, L. N., Walker, T. J., Ebunlomo, E., Ricks, K. B., Prasad, S., Savas, L. S., & Fernandez, M. E. (2022). Using Implementation Mapping to Develop Implementation Strategies for the Delivery of a Cancer Prevention and Control Phone Navigation Program: A Collaboration With 2-1-1. Health Promot Pract, 23(1), 86-97. https://doi.org/10.1177/1524839920957979

Jager, C., Freund, T., Steinhauser, J., Aakhus, E., Flottorp, S., Godycki-Cwirko, M., van Lieshout, J., Krause, J., Szecsenyi, J., & Wensing, M. (2014). Tailored Implementation for Chronic Diseases (TICD): a protocol for process evaluation in cluster randomized controlled trials in five European countries. Trials, 15, 87. https://doi.org/10.1186/1745-6215-15-87

Kansal, A., Quinlan, C., Stark, Z., Kerr, P. G., Mallett, A. J., Lakshmanan, C., Best, S., & Jayasinghe, K. (2022). Theory Designed Strategies to Support Implementation of Genomics in Nephrology. Genes (Basel), 13(10). https://doi.org/10.3390/genes13101919

Kernan, L. M., Dryden, E. M., Nearing, K., Kennedy, M. A., Hung, W., Moo, L., & Pimentel, C. B. (2023). Integrating CFIR-ERIC and e-Delphi Methods to Increase Telegeriatrics Uptake. Gerontologist, 63(3), 545-557. https://doi.org/10.1093/geront/gnac107

Kirk, J. W., Nilsen, P., Andersen, O., Powell, B. J., Tjornhoj-Thomsen, T., Bandholm, T., & Pedersen, M. M. (2022). Co-designing implementation strategies for the WALK-Cph intervention in Denmark aimed at increasing mobility in acutely hospitalized older patients: a qualitative analysis of selected strategies and their justifications. BMC Health Serv Res, 22(1), 8. https://doi.org/10.1186/s12913-021-07395-z

Kurlander, J. E., Helminski, D., Lanham, M., Henstock, J. L., Kidwell, K. M., Krein, S. L., Saini, S. D., Richardson, C. R., De Vries, R., Resnicow, K., Ruff, A. L., Wallace, D. M., Jones, E. K., Perry, L. K., Parsons, J., Ha, N., Alexandris-Souphis, T., Dedrick, D., Aldridge, E., & Barnes, G. D. (2022). Development of a multicomponent implementation strategy to reduce upper gastrointestinal bleeding risk in patients using warfarin and antiplatelet therapy, and protocol for a pragmatic multilevel randomized factorial pilot implementation trial. Implement Sci Commun, 3(1), 8. https://doi.org/10.1186/s43058-022-00256-8

Kwok, E. Y. L., Moodie, S. T. F., Cunningham, B. J., & Oram Cardy, J. E. (2020). Selecting and tailoring implementation interventions: a concept mapping approach. BMC Health Serv Res, 20(1), 385. https://doi.org/10.1186/s12913-020-05270-x

Li, J., Smyth, S. S., Clouser, J. M., McMullen, C. A., Gupta, V., & Williams, M. V. (2021). Planning Implementation Success of Syncope Clinical Practice Guidelines in the Emergency Department Using CFIR Framework. Medicina (Kaunas), 57(6). https://doi.org/10.3390/medicina57060570

McArthur, C., Ziebart, C., Papaioannou, A., Cheung, A. M., Laprade, J., Lee, L., Jain, R., & Giangregorio, L. M. (2018). "We get them up, moving, and out the door. How do we get them to do what is recommended?" Using behaviour change theory to put exercise evidence into action for rehabilitation professionals. Arch Osteoporos, 13(1), 7. https://doi.org/10.1007/s11657-018-0419-7

Midboe, A. M., Martino, S., Krein, S. L., Frank, J. W., Painter, J. T., Chandler, M., Schroeder, A., Fenton, B. T., Troszak, L., Erhardt, T., Kerns, R. D., & Becker, W. C. (2018). Testing implementation facilitation of a primary care-based collaborative care clinical program using a hybrid type III interrupted time series design: a study protocol. Implement Sci, 13(1), 145. https://doi.org/10.1186/s13012-018-0838-2

Mills, S. L., Bergeron, K., & Perez, G. (2015). Using Concept Mapping to Develop a Strategy for Self-Management Support for Underserved Populations Living With Chronic Conditions, British Columbia, August 2013-June 2014. Prev Chronic Dis, 12, E173. https://doi.org/10.5888/pcd12.150183

Moise, N., Phillips, E., Carter, E., Alcantara, C., Julian, J., Thanataveerat, A., Schwartz, J. E., Ye, S., Duran, A., Shimbo, D., & Kronish, I. M. (2020). Design and study protocol for a cluster randomized trial of a multi-faceted implementation strategy to increase the uptake of the USPSTF hypertension screening recommendations: the EMBRACE study. Implement Sci, 15(1), 63. https://doi.org/10.1186/s13012-020-01017-8

Moore, J. E., Mascarenhas, A., Marquez, C., Almaawiy, U., Chan, W. H., D'Souza, J., Liu, B., Straus, S. E., & Team, M. O. (2014). Mapping barriers and intervention activities to behaviour change theory for Mobilization of Vulnerable Elders in Ontario (MOVE ON), a multi-site implementation intervention in acute care hospitals. Implement Sci, 9, 160. https://doi.org/10.1186/s13012-014-0160-6

Munir, F., Kalawsky, K., Wallis, D. J., & Donaldson-Feilder, E. (2013). Using intervention mapping to develop a work-related guidance tool for those affected by cancer. BMC Public Health, 13, 6. https://doi.org/10.1186/1471-2458-13-6

O'Grady, M. A., Kapoor, S., Harrison, L., Kwon, N., Suleiman, A. O., & Muench, F. J. (2022). Implementing a text-messaging intervention for unhealthy alcohol use in emergency departments: protocol for implementation strategy development and a pilot cluster randomized implementation trial. Implement Sci Commun, 3(1), 86. https://doi.org/10.1186/s43058-022-00333-y

Perez Jolles, M., Fernandez, M. E., Jacobs, G., De Leon, J., Myrick, L., & Aarons, G. A. (2022). Using Implementation Mapping to develop protocols supporting the implementation of a state policy on screening children for Adverse Childhood Experiences in a system of health centers in inland Southern California. Front Public Health, 10, 876769. https://doi.org/10.3389/fpubh.2022.876769

Piat, M., Sofouli, E., Wainwright, M., Albert, H., Rivest, M. P., Casey, R., LeBlanc, S., Labonte, L., O'Rourke, J. J., & Kasdorf, S. (2022). Translating mental health recovery guidelines into recovery-oriented innovations: A strategy combining implementation teams and a facilitated planning process. Eval Program Plann, 91, 102054. https://doi.org/10.1016/j.evalprogplan.2022.102054

Podolak, I., Kisia, C., Omosa-Manyonyi, G., & Cosby, J. (2017). Using a multimethod approach to develop implementation strategies for a cervical self-sampling program in Kenya. BMC Health Serv Res, 17(1), 222. https://doi.org/10.1186/s12913-017-2160-0

Powell, B. J., Beidas, R. S., Lewis, C. C., Aarons, G. A., McMillen, J. C., Proctor, E. K., & Mandell, D. S. (2017). Methods to Improve the Selection and Tailoring of Implementation Strategies [10.1007/s11414-015-9475-6]. J Behav Health Serv Res, 44(2), 177-194. https://doi.org/10.1007/s11414-015-9475-6

Powell, B. J., Haley, A. D., Patel, S. V., Amaya-Jackson, L., Glienke, B., Blythe, M., Lengnick-Hall, R., McCrary, S., Beidas, R. S., Lewis, C. C., Aarons, G. A., Wells, K. B., Saldana, L., McKay, M. M., & Weinberger, M. (2020). Improving the implementation and sustainment of evidence-based practices in community mental health organizations: a study protocol for a matched-pair cluster randomized pilot study of the Collaborative Organizational Approach to Selecting and Tailoring Implementation Strategies (COAST-IS). Implement Sci Commun, 1. https://doi.org/10.1186/s43058-020-00009-5

Reuter, K., Genao, K., Callanan, E. M., Cannone, D. E., Giardina, E. G., Rollman, B. L., Singer, J., Slutzky, A. R., Ye, S., Duran, A. T., & Moise, N. (2022). Increasing Uptake of Depression Screening and Treatment Guidelines in Cardiac Patients: A Behavioral and Implementation Science Approach to Developing a Theory-Informed, Multilevel Implementation Strategy. Circ Cardiovasc Qual Outcomes, 15(11), e009338. https://doi.org/10.1161/CIRCOUTCOMES.122.009338

Riordan, F., Racine, E., Phillip, E. T., Bradley, C., Lorencatto, F., Murphy, M., Murphy, A., Browne, J., Smith, S. M., Kearney, P. M., & McHugh, S. M. (2020). Development of an intervention to facilitate implementation and uptake of diabetic retinopathy screening. Implement Sci, 15(1), 34. https://doi.org/10.1186/s13012-020-00982-4

Riphagen-Dalhuisen, J., Frijstein, G., van der Geest-Blankert, N., Danhof-Pont, M., de Jager, H., Bos, N., Smeets, E., de Vries, M., Gallee, P., & Hak, E. (2013). Planning and process evaluation of a multi-faceted influenza vaccination implementation strategy for health care workers in acute health care settings. BMC Infect Dis, 13, 235. https://doi.org/10.1186/1471-2334-13-235

Robitaille, E., MacRae, M., Rowe, P., & Aiken, A. B. (2019). A knowledge translation implementation strategy to promote evidence-based practices in the management of lateral ankle sprains by Canadian Armed Forces physiotherapists. Journal of Military, Veteran and Family Health, 5(2), 50-59. https://doi.org/10.3138/jmvfh.2018-0041

Rogal, S. S., Yakovchenko, V., Morgan, T., Bajaj, J. S., Gonzalez, R., Park, A., Beste, L., Miech, E. J., Lamorte, C., Neely, B., Gibson, S., Malone, P. S., Chartier, M., Taddei, T., Garcia-Tsao, G., Powell, B. J., Dominitz, J. A., Ross, D., & Chinman, M. J. (2020). Getting to implementation: a protocol for a Hybrid III stepped wedge cluster randomized evaluation of using data-driven implementation strategies to improve cirrhosis care for Veterans. Implement Sci, 15(1), 92. https://doi.org/10.1186/s13012-020-01050-7

Ross, J., Stevenson, F., Dack, C., Pal, K., May, C., Michie, S., Barnard, M., & Murray, E. (2018). Developing an implementation strategy for a digital health intervention: an example in routine healthcare. BMC Health Serv Res, 18(1), 794. https://doi.org/10.1186/s12913-018-3615-7

Roth, I. J., Tiedt, M. K., Barnhill, J. L., Karvelas, K. R., Faurot, K. R., Gaylord, S., Gardiner, P., Miller, V. E., & Leeman, J. (2021). Feasibility of Implementation Mapping for Integrative Medical Group Visits. J Altern Complement Med, 27(S1), S71-S80. https://doi.org/10.1089/acm.2020.0393

Rusch, A., DeCamp, L. M., Liebrecht, C. M., Choi, S. Y., Dalack, G. W., Kilbourne, A. M., & Smith, S. N. (2021). A Roadmap to Inform the Implementation of Evidence-Based Collaborative Care Interventions in Communities: Insights From the Michigan Mental Health Integration Partnership. Front Public Health, 9, 655999. https://doi.org/10.3389/fpubh.2021.655999

Shrubsole, K., Copland, D., Hill, A., Khan, A., Lawrie, M., O’Connor, D. A., Pattie, M., Rodriguez, A., Ward, E. C., Worrall, L., & McSween, M.-P. (2022). Development of a tailored intervention to implement an Intensive and Comprehensive Aphasia Program (ICAP) into Australian health services. Aphasiology, 37(9), 1386-1409. https://doi.org/10.1080/02687038.2022.2095608

Simpson, K. M., Porter, K., McConnell, E. S., Colon-Emeric, C., Daily, K. A., Stalzer, A., & Anderson, R. A. (2013). Tool for evaluating research implementation challenges: a sense-making protocol for addressing implementation challenges in complex research settings. Implement Sci, 8, 2. https://doi.org/10.1186/1748-5908-8-2

Sinnema, H., Majo, M. C., Volker, D., Hoogendoorn, A., Terluin, B., Wensing, M., & van Balkom, A. (2015). Effectiveness of a tailored implementation programme to improve recognition, diagnosis and treatment of anxiety and depression in general practice: a cluster randomised controlled trial. Implementation Science, 10(1), 33. https://doi.org/10.1186/s13012-015-0210-8

Stewart, R. E., Beidas, R. S., Last, B. S., Hoskins, K., Byeon, Y. V., Williams, N. J., & Buttenheim, A. M. (2021). Applying NUDGE to Inform Design of EBP Implementation Strategies in Community Mental Health Settings. Adm Policy Ment Health, 48(1), 131-142. https://doi.org/10.1007/s10488-020-01052-z

Teachout, E., Rowe, L. A., Pachon, H., Tsang, B. L., Yeung, L. F., Rosenthal, J., Razzaghi, H., Moore, M., Panagides, D., Milani, P., & Cannon, M. J. (2021). Systematic Process Framework for Conducting Implementation Science Research in Food Fortification Programs. Glob Health Sci Pract, 9(2), 412-421. https://doi.org/10.9745/GHSP-D-20-00707

van Noort, H. H. J., Heinen, M., van Asseldonk, M., Ettema, R. G. A., Vermeulen, H., Huisman-de Waal, G., & On the behalf of the Basic Care Revisited Research, g. (2020). Using intervention mapping to develop an outpatient nursing nutritional intervention to improve nutritional status in undernourished patients planned for surgery. BMC Health Serv Res, 20(1), 152. https://doi.org/10.1186/s12913-020-4964-6

van Oers, H. A., Teela, L., Schepers, S. A., Grootenhuis, M. A., Haverman, L., PROMs, I., & Group, P. R. i. C. P. I. S. (2021). A retrospective assessment of the KLIK PROM portal implementation using the Consolidated Framework for Implementation Research (CFIR). Qual Life Res, 30(11), 3049-3061. https://doi.org/10.1007/s11136-020-02586-3

van Sluisveld, N., Zegers, M., Westert, G., van der Hoeven, J. G., & Wollersheim, H. (2013). A strategy to enhance the safety and efficiency of handovers of ICU patients: study protocol of the pICUp study. Implement Sci, 8, 67. https://doi.org/10.1186/1748-5908-8-67

Versluis, A., van Luenen, S., Meijer, E., Honkoop, P. J., Pinnock, H., Mohr, D. C., Neves, A. L., Chavannes, N. H., & van der Kleij, R. (2020). SERIES: eHealth in primary care. Part 4: Addressing the challenges of implementation. Eur J Gen Pract, 26(1), 140-145. https://doi.org/10.1080/13814788.2020.1826431

Waltz, T. J., Powell, B. J., Fernandez, M. E., Abadie, B., & Damschroder, L. J. (2019). Choosing implementation strategies to address contextual barriers: diversity in recommendations and future directions. Implement Sci, 14(1), 42. https://doi.org/10.1186/s13012-019-0892-4

Watkins, K. E., Ober, A. J., Lamp, K., Lind, M., Diamant, A., Osilla, K. C., Heinzerling, K., Hunter, S. B., & Pincus, H. A. (2017). Implementing the Chronic Care Model for Opioid and Alcohol Use Disorders in Primary Care. Prog Community Health Partnersh, 11(4), 397-407. https://doi.org/10.1353/cpr.2017.0047

Wensing, M. (2017). The Tailored Implementation in Chronic Diseases (TICD) project: introduction and main findings. Implement Sci, 12(1), 5. https://doi.org/10.1186/s13012-016-0536-x

Wilkinson, S. A., Hickman, I., Cameron, A., Young, A., Olenski, S., PM, B. P., & O'Brien, M. (2022). 'It seems like common sense now': experiences of allied health clinicians participating in a knowledge translation telementoring program. JBI Evid Implement, 20(3), 189-198. https://doi.org/10.1097/XEB.0000000000000297

Wilson, N. A., Tcheng, J. E., Graham, J., & Drozda, J. P., Jr. (2022). Advancing Patient Safety Surrounding Medical Devices: Barriers, Strategies, and Next Steps in Health System Implementation of Unique Device Identifiers. Med Devices (Auckl), 15, 177-186. https://doi.org/10.2147/MDER.S364539

Zhao, J., Harvey, G., Vandyk, A., & Gifford, W. (2022). Social Media for ImpLementing Evidence (SMILE): Conceptual Framework. JMIR Form Res, 6(3), e29891. https://doi.org/10.2196/29891
